# Innovations in Alzheimer’s disease diagnostic technologies: clinical prospects of novel biomarkers, multimodal integration, and non-invasive detection

**DOI:** 10.3389/fneur.2025.1651708

**Published:** 2025-10-29

**Authors:** Rui Wang, Siqin Peng, Jianrong Zhu, Ye Xu, Minghao Wang, Ling Zhang, Yilan Qiu, Defu Hou, Qinglin Wang, Rushi Liu

**Affiliations:** ^1^College of Medical Technology and Translational Medicine, Hunan Normal University, Changsha, China; ^2^Hunan Xuxiang Biotechnology Co., Ltd., Changsha, China; ^3^School of Life Sciences, Hunan Normal University, Changsha, China

**Keywords:** Alzheimer’s disease diagnosis, β-amyloid, tau protein phosphorylation, non-invasive biomarkers, multimodal diagnostic technologies

## Abstract

Alzheimer's disease (AD) is a neurodegenerative disorder characterized by the deposition of β-amyloid (Aβ) plaques and the formation of neurofibrillary tangles composed of hyperphosphorylated tau protein, ultimately leading to cognitive decline and neuronal loss. Current diagnostic methods, including clinical evaluations, neuroimaging examinations, and cerebrospinal fluid biomarker testing, face challenges such as insufficient sensitivity and specificity, as well as operational complexity. In recent years, significant advancements have been made in diagnostic technologies, with the emergence of new biomarkers and detection methods, including blood-based Aβ and tau protein detection, ocular biomarker testing, and non-invasive screening through urine or breath analysis. These innovative developments, combined with multimodal diagnostic technologies that integrate imaging, genomics, and proteomics, have opened new possibilities for the early diagnosis and precise staging of Alzheimer's disease. Furthermore, advancements in microfluidic chips and biosensor technologies have enhanced the capability for rapid, efficient, and cost-effective diagnosis. As research continues to evolve, the gradual application of these advanced technologies in clinical practice is expected to revolutionize the management of Alzheimer's disease, facilitating early intervention and the formulation of individualized treatment strategies.

## 1 Introduction

Alzheimer’s Disease (AD) is a chronic neurodegenerative disorder that predominantly affects neurons in the brain, resulting in the progressive deterioration of memory, cognition, and behavior. The disease imposes significant psychological and financial burdens on both patients and their families (1). Currently, the treatment of AD primarily aims to alleviate symptoms and enhance the quality of life for affected individuals, as there is no known cure. Globally, the incidence and prevalence of AD are increasing. According to Alzheimer’s Disease International, over 50 million people worldwide were living with Alzheimer’s disease in 2020, and this number is projected to rise to 150 million by 2050. The pathological changes associated with AD primarily include age-related plaques formed by the deposition of beta-amyloid protein (Aβ), neurofibrillary tangles resulting from the hyperphosphorylation of tau protein, and glial cell hyperplasia accompanied by neuronal loss. Currently, the diagnosis of AD primarily relies on clinical manifestations, neuropsychological assessments, and imaging techniques such as cerebrospinal fluid analysis, PET, and MRI (2, 3). However, the current *in vitro* diagnosis of AD faces multiple challenges, including the following: the complexity of pathological markers. In 2024, plasma biomarkers were incorporated into the diagnostic criteria, facilitating a more accessible and cost-effective diagnosis. The core pathological features of AD are the presence of Aβ plaques and neurofibrillary tangles of tau protein. Abnormal deposits of these pathological markers are critical for diagnosis, but they can present variably across different types of Alzheimer’s disease, leading to diagnostic uncertainty. Additionally, while the detection of biomarkers such as Aβ and tau proteins has demonstrated some sensitivity and specificity in clinical trials, these metrics are not consistently optimal. For instance, a positive biomarker result may not accurately indicate AD in certain cases, and significant pathological changes may go undetected in some patients. The early stages of AD are often characterized by mild cognitive decline, complicating the diagnostic process. Presently, most diagnostic tests concentrate on the later stages of the disease, when symptoms are more pronounced, thereby missing the crucial opportunity for early diagnosis. Furthermore, while advanced *in vitro* diagnostic techniques such as PET and cerebrospinal fluid testing can yield detailed biomarker information for AD, their accessibility and cost may hinder widespread implementation in clinical practice (1, 4, 5).

In summary, *in vitro* diagnosis of Alzheimer’s disease faces multiple challenges, which need to take into account factors such as the complexity of pathological markers, the accuracy of diagnostic techniques, the feasibility of early diagnosis, and the accessibility and cost of techniques. In the future, with the deepening of research and the advancement of technology, it is expected to improve the diagnostic accuracy and efficiency of Alzheimer’s disease.

## 2 Pathophysiological mechanism of Alzheimer’s disease

### 2.1 Amyloid hypothesis

In the field of Alzheimer’s disease (AD), the “amyloid cascade hypothesis” used to be the main theory of the pathogenesis of AD (6), which posits that Aβ deposition is the initial event in the pathogenesis of AD, leading to the formation of tau tangles, loss of neurons, dysfunction and cognitive decline (7). However, this hypothesis has been called into question due to the repeated failure of clinical trials targeting Aβ.

Aβ is produced through the hydrolysis of amyloid precursor protein (APP) by beta- and gamma-secretase enzymes, and it exhibits neurotoxicity (8). Under normal circumstances, Aβ is cleared by the metabolic system; however, in patients with AD, there exists an imbalance between its production and clearance, resulting in a gradual accumulation in the brain. Aβ can aggregate into various forms, including monomers, oligomers, fibrils, and mature amyloid plaques, with oligomers being the most neurotoxic. These oligomers can directly induce hyperphosphorylation of tau protein and lead to neurodegenerative changes, closely correlating with cognitive function impairment and the pathological alterations associated with AD (9–11). The aggregation of Aβ is influenced by several factors, including its concentration, amino acid sequence, pH, ionic strength, and the presence of metal ions such as copper, zinc, and iron, which can promote Aβ aggregation. Furthermore, neuronal insulin signaling is implicated in Aβ dynamics, as insulin signaling within the central nervous system can prevent the accumulation of Aβ oligomers (AβO) and inhibit their neurotoxic binding (12). However, AβO can also disrupt insulin signaling by inhibiting the key effector IRS-1, thereby obstructing the transport of insulin receptors to dendritic membranes and impairing insulin signaling in central nervous system neurons (13).

### 2.2 Abnormal tau protein

#### 2.2.1 Relationship between tau protein hyperphosphorylation and DEK protein

DEK is a chromatin remodeling nuclear protein associated with DNA replication and repair, cell proliferation, and apoptosis inhibition. Loss of DEK expression leads to overexpression and hyperphosphorylation of Tau protein. The specific mechanism may be that DEK protein is normally involved in certain cellular pathways and plays a regulatory role in the phosphorylation level of Tau protein. When DEK is absent, this regulatory mechanism is disrupted, resulting in Tau hyperphosphorylation (14).

#### 2.2.2 Relationship with protein phosphatases and kinases

Cerebral ischemia may lead to abnormal regulation of protein kinase and phosphatase, resulting in hyperphosphorylation of Tau protein. For example, in mouse models of cerebral ischemia, ischemia activates the lysosomal enzyme asparagine endopeptidase (AEP). AEP cleaved 2 (I2PP2A), an inhibitor of protein phosphatase 2A (PP2A), allowing I2PP2A to transfer from the nucleus of neurons into the cytoplasm. This process leads to hyperphosphorylation of Tau protein by inhibiting PP2A (15).

#### 2.2.3 Relationship with glycogen synthase kinase-3β (GSK-3β)

Inhibition of abnormal phosphorylation (hyperphosphorylation) during the fibrillation of tau protein may impede the formation of neurofibrillary tangles. Glycogen synthase kinase 3 beta (GSK-3β) is believed to play a significant role in the formation of neurofibrillary tangles. Currently, studying animal models is considered essential for elucidating the mechanisms underlying the formation of neurofibrillary tangles in Alzheimer’s disease; however, the specific mechanism of action of GSK-3β in this process requires further investigation (16).

#### 2.2.4 Tau hyperphosphorylation and the relationship between neurofibrillary tangles and AD

The interaction between Tau and the nuclear pore complex (NPC) is significant and cannot be overlooked. Tau directly interacts with nuclear pore proteins, thereby influencing the structural and functional integrity of the NPC. In pathological conditions, Tau can disrupt nuclear transport processes, resulting in the accumulation of the nuclear pore protein Nup98 in certain neurons within the cell body. This accumulation may subsequently promote Tau aggregation, potentially contributing to the formation of neurofibrillary tangles (17, 18).

Neurofibrillary tangles are primarily caused by excessive phosphorylation of tau protein. Microglia can exacerbate the inflammatory response by releasing various inflammatory mediators, which in turn affects tau protein phosphorylation and the formation of neurofibrillary tangles. The impact on cognitive decline: The deposition of neurofibrillary tangles is closely associated with cognitive decline in AD. Hyperphosphorylation of tau protein and the formation of paired helical filaments are believed to underlie neuronal degeneration in this condition. In the pathogenesis of Alzheimer’s disease, alterations in tau protein may occur independently of the cascade reaction initiated by *β*-amyloid deposition. Furthermore, clinical trials have confirmed the efficacy of tau-related vaccine immunization (19).

### 2.3 Neuroinflammation and oxidative stress

#### 2.3.1 Neuroinflammation

In AD, neuroinflammation is primarily triggered by Aβ deposition and tau hyperphosphorylation. Aβ aggregates activate microglia and astrocytes, which are key players in the neuroinflammatory response within the central nervous system. For instance, once Aβ plaques form in the brain, microglia recognize and engulf Aβ, a process that leads to microglial activation and the release of numerous inflammatory mediators (20, 21). Activated microglia and astrocytes secrete various inflammatory mediators, including cytokines such as tumor necrosis factor-alpha (TNF-*α*), interleukin-1beta (IL-1β), and interleukin-6 (IL-6). Studies indicate that these cytokines can exert direct toxic effects on neurons, impacting neuronal survival, synaptic plasticity, and neurotransmitter metabolism. For example, TNF-*α* can induce neuronal apoptosis and disrupt nerve signaling by interfering with the normal release and uptake of neurotransmitters (22). Concurrently, neuroinflammation is associated with increased oxidative stress. Reactive oxygen species (ROS) and reactive nitrogen species (RNS) produced during inflammation can further damage neuronal cell membranes, mitochondria, and other cellular structures. For instance, nitric oxide (NO) released following microglial activation can react with superoxide anions to produce peroxynitrite at elevated concentrations, a potent oxidizing agent that can cause oxidative damage to proteins, lipids, and DNA in neurons (23).

**Figure 1:**
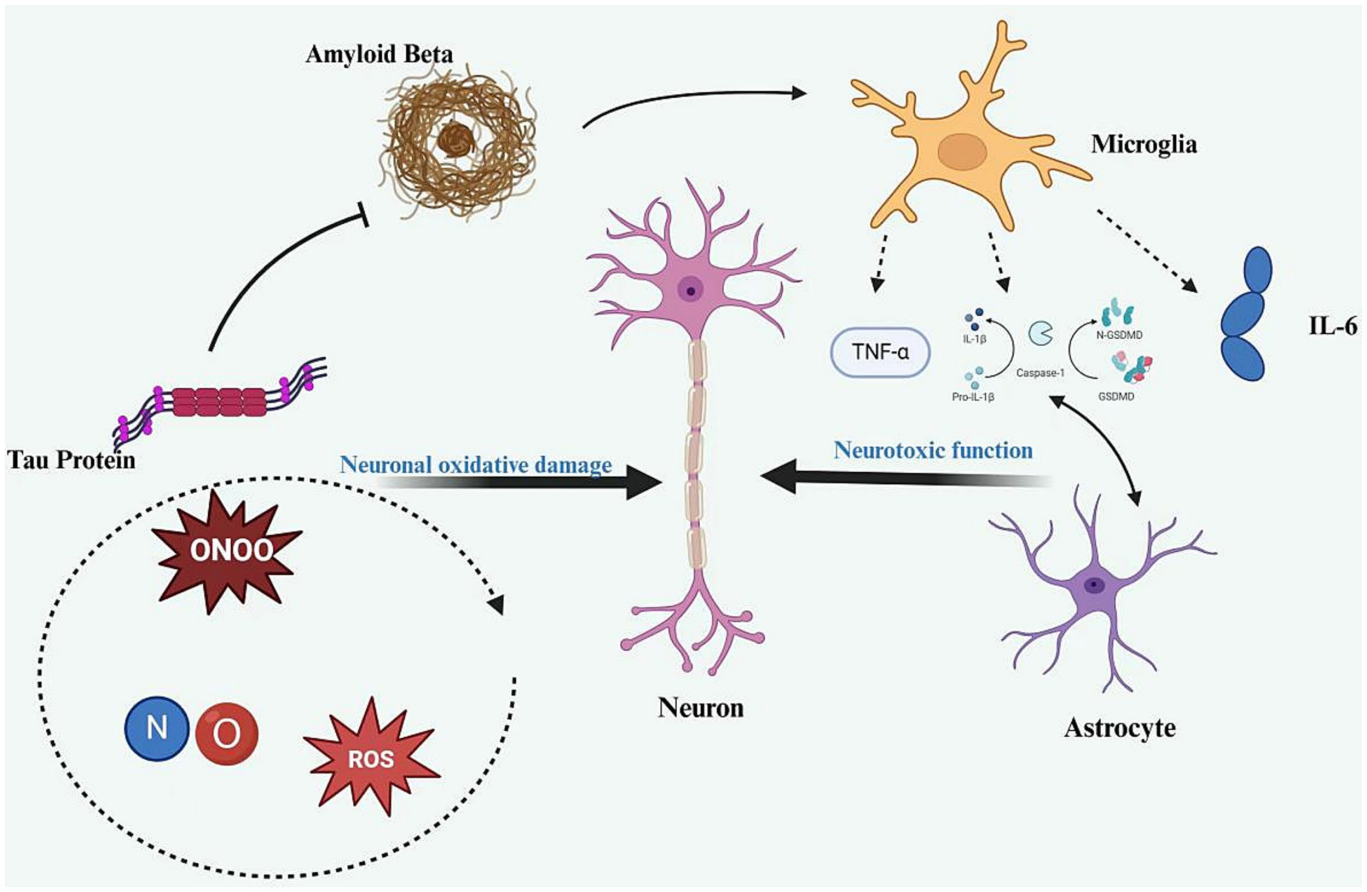
The mechanism of neuroinflammation in AD.

Neuroinflammation establishes a vicious cycle that accelerates the progression of AD. On one hand, neuronal damage resulting from the inflammatory response promotes further Aβ production and hyperphosphorylation of tau proteins. For example, after neuronal injury, substances may be released that enhance the accumulation and deposition of Aβ. On the other hand, a sustained inflammatory response recruits additional immune cells, broadens the inflammatory response, and propagates neuroinflammation, leading to further neuronal damage (24–26) (Figure 1).

#### 2.3.2 Oxidative stress

The mechanisms of oxidative stress include mitochondrial dysfunction and metal ion imbalance. Among them, mitochondrial dysfunction is often shown as abnormal in the brain of AD patients. Mitochondria are the main energy-producing sites in cells and one of the main sources of reactive oxygen species (ROS). When the mitochondrial respiratory chain is damaged, the electron transport process will leak, resulting in the production of large amounts of ROS (27, 28). For example, Aβ can bind directly to mitochondria, interfering with their normal function and causing them to produce too many ROS. In terms of metal ion imbalance, metal ions such as iron, copper, and zinc play an important role in REDOX reactions in the brain. In the brains of people with AD, the balance of these metal ions is disrupted. For example, an abnormal accumulation of iron ions produces large quantities of hydroxyl radicals (· OH) through the Fenton reaction, a highly reactive ROS that can trigger lipid peroxidation, protein oxidation, and DNA damage (29, 30).

Biomarkers associated with oxidative stress include oxidative products and antioxidant enzymes. MDA, the end product of lipid peroxidation in oxidative products, is often elevated in the blood, cerebrospinal fluid and brain tissue of patients with AD. In addition, protein oxidation products such as carbonyl protein and DNA oxidative damage product 8-hydroxy-deoxyguanosine (8-OHdG) can also be used as markers of oxidative stress (31). For example, in the brain tissue of AD patients, the content of carbonyl protein is significantly higher than that of the normal population, indicating oxidative damage to proteins within neurons (32).

In terms of oxidases, SOD, glutathione peroxidase (GSH-Px), and catalase (CAT) are crucial intracellular antioxidant enzymes. In patients with AD, the activity of these antioxidant enzymes may be altered. For instance, some studies have indicated that SOD activity may increase in the brains of AD patients, potentially serving as a compensatory response to oxidative stress; however, this compensatory mechanism may gradually fail as the disease progresses (33). Furthermore, studies have identified the light chain of NFL as a marker for neuronal axon damage. In AD patients, NFL levels are significantly elevated due to neuronal damage resulting from neuroinflammation and oxidative stress. NFL can serve as an indicator to assess the progression of AD and the extent of neuronal damage. For example, in the cerebrospinal fluid of AD patients, NFL concentrations have been positively correlated with the degree of cognitive decline (34, 35).

### 2.4 Other pathologic mechanisms

The pathogenesis of AD involves A variety of pathological processes. Besides the two main features of Aβ deposition and Tau protein hyperphosphorylation, it also includes mechanisms such as neuron loss, synaptic dysfunction and mitochondrial dysfunction (36).

#### 2.4.1 Loss of neurons

Widespread loss of neurons in the brain primarily occurs in the hippocampal and cortical regions, resulting in a significant decline in memory and cognitive function. This neuronal loss is strongly associated with various factors, including the toxic effects caused by Aβ deposition and oxidative stress. Collectively, these factors contribute to neuronal dysfunction and death (37). In 2023, a research team at the University of Leuven in Belgium utilized a human-mouse chimeric brain model and discovered that the long non-coding RNA MEG3 specifically induces necrotic cell death in grafts of human neurons, leading to neuronal loss characteristic of Alzheimer’s disease. This study demonstrated that amyloid plaque pathology is sufficient to induce tau pathology, neuronal death, and other core features of Alzheimer’s disease in human neurons, suggesting the presence of human-specific factors that render human neurons more sensitive to amyloid plaques. Mechanistic studies revealed that long-chain non-coding RNA MEG3 mediates this process, being specifically upregulated in human neurons and leading to neuronal loss through the activation of the RIPK1/RIPK3/MLKL necrosis pathway. Targeting this necrotic pathway is anticipated to be a novel strategy for treating Alzheimer’s disease (38).

#### 2.4.2 Synaptic dysfunction

Synaptic dysfunction is mainly manifested by decreased synaptic plasticity and decreased synaptic density. Synaptic plasticity is the basis of learning and memory, and its impairment can lead to significant decline in cognitive ability (39). Studies have shown that deposition of Aβ and hyperphosphorylation of Tau protein are strongly associated with synaptic dysfunction. The oligomers of Aβ can be directly toxic to synapses, affecting the plasticity and stability of synapses, thus compromising learning and memory function (40). Recent studies have shown that Aβ induces Jacob’s nucleocytoplasmic transport in the brains of AD patients and in mouse hippocampal neurons. Jacob is a protein that connects the NMDA receptor-derived semaphore to CREB. Aβ regulates the transport of Jacob, resulting in the transcriptional inactivation of CREB, which triggers synaptic damage and loss in mouse models of AD. The small compound nitrophenylarsonic acid selectively obstructs the assembly of the Jacob/LIM-only 4 (LMO4)/protein phosphatase 1 (PP1) semaphone, thereby restoring CREB transcriptional activity. Nitrophenylarsonic acid has been shown to prevent synaptic plasticity damage and cognitive decline in mouse models of AD. Taken together, these data suggest that targeting Jacob protein-induced CREB inactivation may serve as a therapeutic pathway against early synaptic dysfunction in AD (41). Together, these pathological changes lead to decreased synaptic transmission efficiency and impaired information exchange between neurons, ultimately leading to cognitive impairment (42).

#### 2.4.3 Mitochondrial dysfunction

Mitochondria play a key role in energy metabolism, and their dysfunction will aggravate neuronal damage. In AD patients, the structure of mitochondria in the brain is abnormal, resulting in impaired energy metabolism and further accelerating nerve cell apoptosis (43). Studies have shown that VDAC1 is a multifunctional protein that is expressed in mitochondria and other cellular compartments, including the plasma membrane. This protein regulates major metabolic and energy functions of cells, including Ca2 + homeostasis, oxidative stress, and mitochondria-mediated apoptosis. Repairing mitochondrial dysfunction by targeting VDAC1 to block its pro-apoptotic activity may represent a novel strategy to inhibit cell death (44).

**Figure 2:**
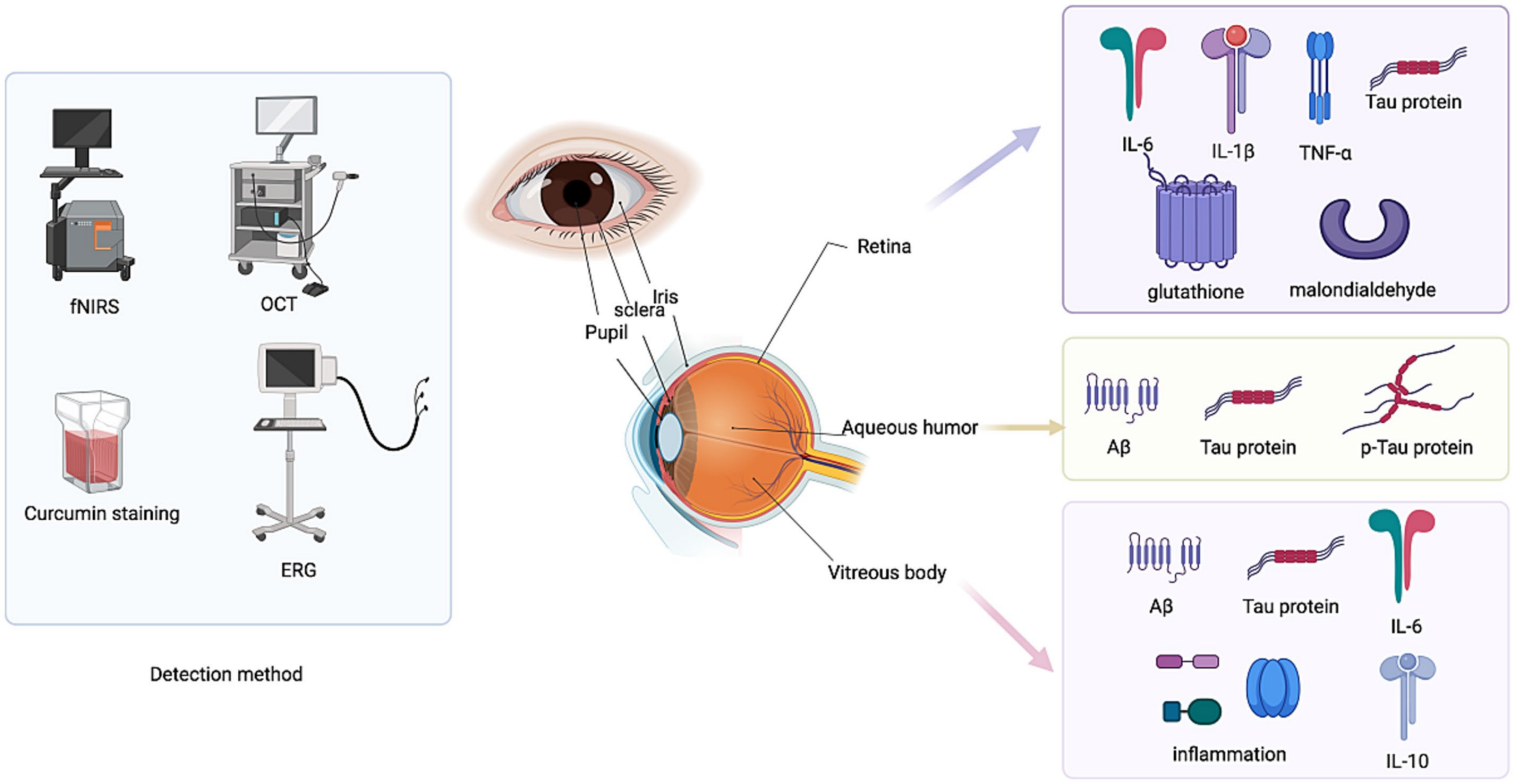
Ocular biomarkers in AD.

In conclusion, the pathogenesis of Alzheimer’s disease is complex and diverse, involving multiple aspects such as neuron loss, synaptic dysfunction, and mitochondrial dysfunction. These mechanisms influence each other and jointly promote the occurrence and development of the disease. In-depth study of these mechanisms will help to provide new ideas and methods for the diagnosis and treatment of AD (Figure 2).

## 3 Traditional *in vitro* diagnostic methods

### 3.1 Neuropsychological evaluation

Scales of neuropsychological evaluation is presented in the Table 1.

**Table 1:** Scales of neuropsychological evaluation.

Evaluation dimension	Item	Method	Purpose	Evaluation	Reference
Cognitive function	Memory	Mini-mental state examination (MMSE)	Classifying subtypes of mild cognitive impairment (MCI) in Alzheimer’s disease, AD, and normal control groups provides significant reference value for early clinical diagnosis, reducing the prevalence of Alzheimer’s disease.	The MMSE scale consists of 30 questions with a score range of 0–30, covering temporal orientation, spatial orientation, immediate memory, attention and calculation, delayed memory, language, and visuospatial abilities. Score ranges: 27–30 normal, 21–26 mild dementia, 10–20 moderate dementia, 0–9 severe dementia.	45
Cognitive function	Memory	Montreal cognitive assessment (MoCA)	Assesses cognitive function, especially for screening mild cognitive impairment, correcting for education level bias	Includes executive function (1 point), fluency (2 points), orientation (6 points), calculation (3 points), abstraction (3 points), delayed recall (5 points), naming (4 points), attention (3 points), visuospatial abilities (3 points), with a total score of 30. For individuals with ≤4 years of education, an additional point is added.	46
Autonomic nervous system function	Autonomic dysfunction	SCOPA-AUT scale assessment	Assess the patient’s autonomic dysfunction to determine the severity of autonomic dysfunction	Includes the digestive system (21 points), urinary system (18 points), temperature (15 points), cardiovascular system (9 points), and sexual function (6 points), with a total score of 69 points. The higher the score, the more severe the autonomic dysfunction.	47
Autonomic nervous function	Determination of autonomic dysfunction	Measurement of orthostatic hypotension (OH); Determination of hyperhidrosis (Body temperature); Assessment of constipation (Digestive system); Evaluation of urinary frequency, urgency, and incomplete emptying (Urinary system); Evaluation of sexual dysfunction (Sexual function)	Determination of the Presence and Duration of Autonomic Dysfunction	OH: BP measured supine/upright with ≥20 mmHg systolic or ≥10 mmHg diastolic drop, or presence of OH symptoms; Hyperhidrosis: Excessive sweating beyond thermoregulatory needs; Constipation: ≤3 bowel movements/week, straining during defecation, or chronic laxative use; Urinary Symptoms: Urgency, daytime frequency, nocturia; Sexual dysfunction: Erectile dysfunction (males) and decreased libido. Scoring: Each symptom = 1 point; symptom duration recorded; absence = 0. Diagnosis: ≥2 symptoms ≥6 months defines autonomic dysfunction.	48

Neuropsychological assessment of AD plays a key role in diagnosing and monitoring disease progression, but there are some shortcomings. For one thing, existing neuropsychological tests may not be sensitive enough in some cases to accurately identify early cognitive impairment. For example, commonly used scales such as the Brief Mental State Examination (MMSE) and the Montreal Cognitive Assessment (MoCA) may have certain limitations in identifying mild cognitive impairment (MCI); On the other hand, the current neuropsychological assessment mainly reflects the changes in cognitive function, but cannot directly reflect the pathophysiological processes of AD, such as neuronal damage and amyloid deposition (45–47).

### 3.2 Imaging tests

#### 3.2.1 Magnetic resonance imaging (MRI)

AD presents as an irreversible and progressive memory dysfunction lasting more than six months (48). MCI is considered an early stage of AD, characterized by atypical clinical manifestations. However, patients diagnosed with both MCI and mild AD may delay cognitive decline through pharmacological interventions, such as Aducanumab (49). Thus, early diagnosis of AD and prognosis of MCI are of paramount importance. In recent years, advancements in biochemistry, genetics, imaging, and other disciplines, coupled with the multidimensional analysis of parameters, have significantly enhanced the accuracy of early diagnosis for both AD and MCI (50, 51). The hippocampus is one of the first regions affected during the progression of AD. Sørensen et al. utilized T1-weighted imaging (T1WI) to extract the texture of the hippocampi, successfully distinguishing between normal aging healthy controls, MCI, and AD patients using the support vector machine (SVM) method (52). The results indicated that the area under the ROC curve (AUC) for healthy controls compared to MCI and healthy controls compared to AD were 0.724 and 0.912, respectively, confirming the efficacy of imaging omics in differentiating normal aging healthy individuals from those with MCI and AD (53). Most studies utilizing MRI-based imaging omics in AD are retrospective analyses, which may introduce variability due to differences in scanning equipment, MRI parameters, and methodologies across medical centers (54). Therefore, establishing standardized medical image data remains an urgent issue to address in current research.

**Figure 3:**
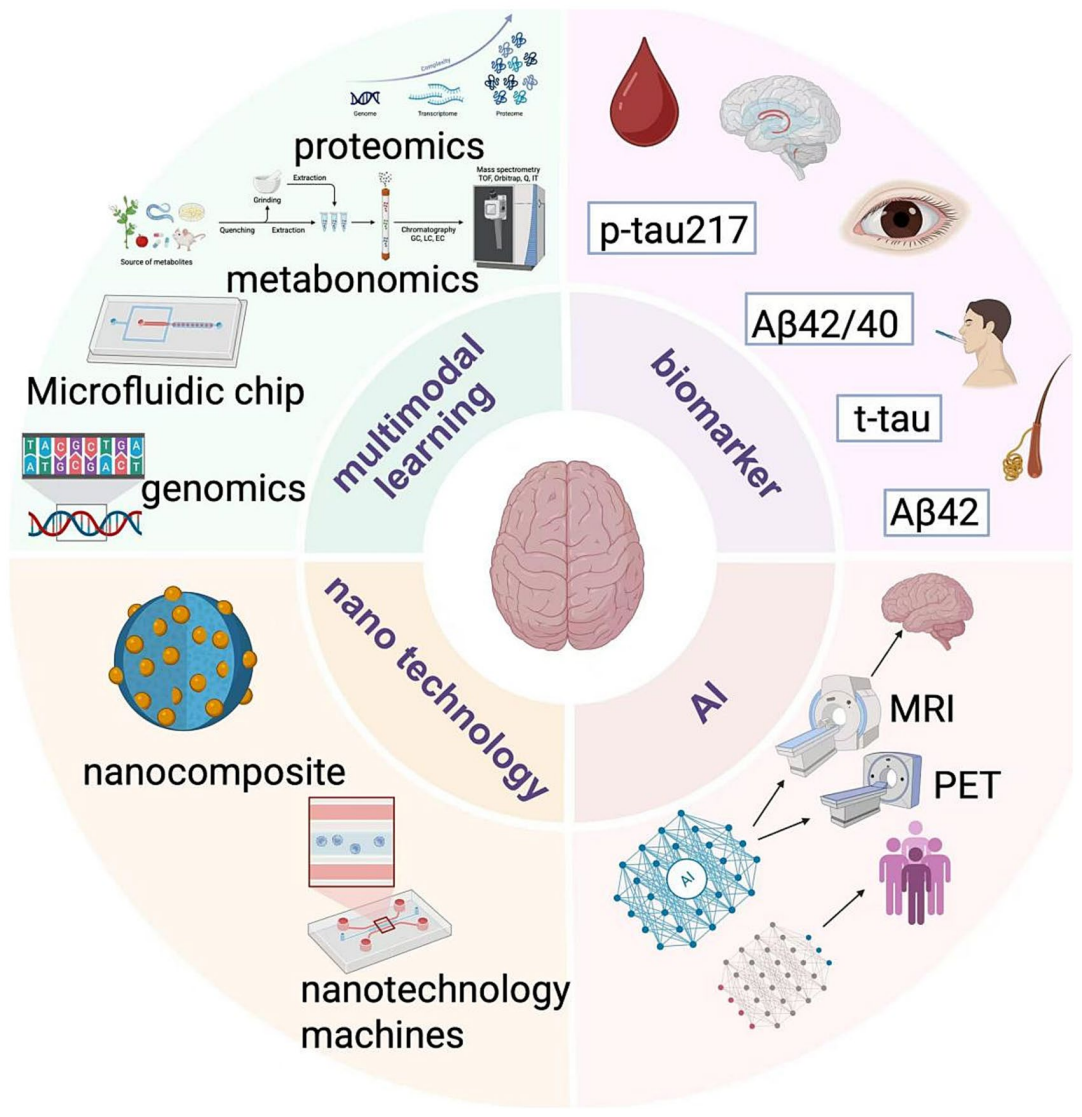
Trends in diagnostic technology.

In clinical practice, the diagnostic sensitivity and specificity of MRI vary according to disease stage. Structural MRI demonstrates approximately 70–80% sensitivity and 65–75% specificity in differentiating MCI from healthy controls, whereas in advanced AD, hippocampal and cortical atrophy increase diagnostic sensitivity to over 90% (55, 56). Functional MRI (fMRI) and diffusion tensor imaging (DTI) have shown improved sensitivity in identifying early microstructural and connectivity changes. Combining MRI-based volumetric analysis with machine learning approaches can increase diagnostic accuracy to over 90% (57). However, inter-scanner variability and lack of standardization remain barriers to cross-study comparison. Therefore, MRI-based multimodal integration with PET or blood biomarkers could enhance early-stage AD identification (Figure 3).

#### 3.2.2 Positron emission tomography (PET)

A*β*-pet is highly sensitive to Alzheimer’s disease and can visualize the distribution and load of Aβ plaques. A positive result indicates the presence of Aβ pathology, while a negative result basically rules out AD. Currently, the US FDA has approved several Aβ-PET radiotracers for the diagnosis of Alzheimer’s disease. Tau-pet can reflect the neurofibrillary tangles of tau protein, which is particularly important in the diagnosis of dementia in Alzheimer’s disease (58). By analyzing image features that are difficult to observe with the naked eye, imaging omics technology has shown good results in a number of studies on the diagnosis and prognosis prediction of AD based on PET images, and also provides a new scheme for objective and repeatable research on AD (59). At present, amyloid PET has been accepted as part of the diagnostic process for AD and is considered as the diagnostic standard for AD in clinical and research use. *In vivo*, the sensitivity and specificity of Aβ deposition in AD patients can reach 96 and 100%, respectively (60). It is important to note that although the amyloid PET can effectively distinguish between most of the AD pathological types of dementia, but hybrid AD and other diseases of the nervous system may also appear obvious amyloid deposits, such as cerebral amyloid angiopathy (cerebral amyloid angiopathy, CAA), some DLB and Parkinson’s disease dementia (PDD), Down’s syndrome, as well as traumatic brain injury and chronic traumatic encephalopathy. Amyloid PET may also present a positive result in these conditions. Therefore, a comprehensive judgment should be made in conjunction with clinical, multimodal imaging and other biomarkers (61).

Although amyloid PET has high sensitivity (up to 96%), its specificity remains questionable in certain clinical settings. A positive amyloid scan does not definitively confirm AD, as amyloid deposition may also appear in conditions such as dementia with Lewy bodies, Parkinson’s disease dementia, and cerebral amyloid angiopathy (62, 63). Therefore, amyloid PET should be interpreted in combination with tau imaging, CSF or plasma biomarkers, and neuropsychological testing to achieve a more accurate diagnosis.

In addition to amyloid PET, Tau PET imaging provides complementary diagnostic value. Tau deposition correlates more closely with cognitive impairment than amyloid burden, allowing better staging of disease progression (64). Studies using 18F-MK-6240 and 18F-AV-1451 tracers report sensitivities above 90% and specificities around 85–90% for differentiating AD from non-AD dementias (65). Nevertheless, false-positive binding can occur in aging or other tauopathies, underscoring the need for multimodal interpretation that includes clinical context and other biomarkers.

Comparative performance of major imaging modalities in AD diagnosis in the Table 2.

**Table 2:** Comparative diagnostic performance of major neuroimaging modalities in AD.

Imaging modality	Biomarker target	Sensitivity (%)	Specificity (%)	Clinical stage applicability	Main strengths	Limitations	Reference
MRI (structural)	Brain atrophy	70–90	65–80	MCI → AD	Widely available, quantitative	Limited early-stage specificity	185
fMRI/DTI	Functional/connectivity	75–85	70–80	Preclinical → MCI	Detects microstructural and functional change	High variability, research use	186
Amyloid PET	Aβ plaques	96	80–90	MCI → AD	Early amyloid detection	False positives in DLB/PDD/CAA	187
Tau PET	Neurofibrillary tangles	90–95	85–90	MCI → AD dementia	Correlates with cognitive severity	Cost, limited tracer availability	188

### 3.3 Cerebrospinal fluid biomarker detection

Recent studies have demonstrated that blood biomarkers for p-tau181, p-tau217, and p-tau231, when integrated with brain tau and Aβ pathophysiology, have been developed and validated (66). Pathological changes in AD can be assessed by analyzing abnormalities in soluble Aβ and tau in CSF, as well as the phosphorylated state of tau. Multiple immunoassays can detect biomarkers such as Aβ, tau proteins, and p-tau in CSF. For instance, several commercial products are available to detect p-tau181 and total tau in CSF (67). The level of variation in p-tau in CSF is highly correlated with that in plasma. In blood, plasma p-tau231, p-tau217, and p-tau181, each measured using N-terminal tau morphology, exhibit similar diagnostic performance and predictive ability regarding brain Aβ and tau, indicating their interchangeability for clinical use. For example, a study published in 2022 found that both p-tau181 and p-tau217 levels in CSF were elevated in a mouse model that overexpressed Aβ or familial Danish dementia amyloid (68). Despite the high acceptability and lower cost of p-tau compared to conventional testing methods, several challenges persist. First, the collection of p-tau is invasive, as a lumbar puncture is required to obtain a sample of CSF. This procedure can cause discomfort and carries risks for the patient, including infection and bleeding. Second, sample collection is challenging; the volume of CSF obtained from a lumbar puncture is often insufficient for measuring multiple AT(N) biomarkers and establishing a biobank, which limits retrospective analysis. Third, ethical challenges arise with the use of p-tau. While the application of p-tau may facilitate AD detection, it significantly increases the risk of disclosing the disease (69, 70).

## 4 Novel *in vitro* diagnostic methods

### 4.1 Blood biomarkers

Since the establishment of the biomarker-based A-T-N (amyloid/Tau/neurodegeneration) framework, the diagnosis of AD has become more precise. Cerebrospinal fluid tests and positron emission tomography tests based on this framework have become widely accepted. However, the A-T-N framework does not cover the full scope of AD pathology, and its invasive nature and high cost limit the application of central nervous system diagnostic methods (70, 71). Some pathological and related biomarkers, such as those related to synaptic damage, neuroinflammation, neuroimmunity, activation of microglia and astrocytes, systemic immunity, systemic inflammation, nutrition and metabolism, apoptosis, mitochondrial dysfunction, and oxidative stress, have not been included in the framework (72). “X” indicates biomarkers for the above or unrealized pathologies, as well as dynamic changes as AD develops. Therefore, adding an “X” to the A-T-N framework to form the A-T-N-X framework can reflect the overall pathological spectrum of AD and shed light on its pathogenesis (73).

#### 4.1.1 Aβ

Aβ is a core biomarker of amyloid plaques in Alzheimer’s disease (AD), with Aβ42 being more specific to AD and Aβ40 serving as the background for total Aβ production. The ratio of Aβ42 to Aβ40 helps to balance basic Aβ production across different individuals. In AD, there is an increase in age-related plaques and a decrease in soluble Aβ42 in cerebrospinal fluid (74, 75). In 2020, C2N Diagnostics launched the mass spectrometric plasma Aβ test (PrecivityAD™), which has been approved in the U.S. and Europe for the diagnosis of AD, demonstrating an 86% agreement (92% sensitivity, 76% specificity) with amyloid PET. Although plasma testing will not completely replace cerebrospinal fluid Aβ and amyloid PET testing, it represents a significant advancement in the field of AD diagnosis (76–78). However, Aβ faces several challenges. For instance, plasma Aβ exhibits a low correlation with cerebrospinal fluid Aβ, particularly when compared to high-precision plasma p-tau. Moreover, Aβ is viscous, and the mechanisms by which it is transported from the brain to the blood remain poorly understood. In cases of blood dilution, CNS-derived soluble Aβ is difficult to detect in plasma, with its levels further diminished during the progression of AD (79). To address these challenges, the research team proposed several solutions. First, it is essential to identify the pathway Aβ takes from the cerebrospinal fluid to the blood and the factors influencing this process. Additionally, the relationships between intracellular Aβ, interstitial Aβ, Aβ in near-death experiences, and Aβ in plasma need to be elucidated. Second, preconditioning before detection can mitigate interference from the complex background of plasma. For example, pre-denaturation enzyme-linked immunosorbent assay (ELISA) can detect released Aβ that is initially captured by various blood proteins (80, 81).

#### 4.1.2 Tau protein

Tau protein is a product of the microtubule associated protein tau (MAPT) gene and plays a physiological role in stabilizing microtubules. In pathological state, tau is the main component of neurofibrillary tangles and is considered to be the downstream protein of Aβ, which can reflect the degree of neuronal damage (82). Post-translational modifications (PTMs) of tau protein include truncation, phosphorylation, acetylation, methylation, ubiquitination, glycosylation, nitrification, etc. These modification sites are associated with pathology, contribute to the diagnosis of AD, and are associated with clinical outcome by affecting the spread or clearance of tau (83). Among them, phosphorylation is the most common PTMS type of tau protein, and hyperphosphorylated tau is a major component of neurofibrillary tangles. For example, P-tau181, P-tau231 and P-tau217 in cerebrospinal fluid and plasma change at the early stage of AD pathology, so they are often used as early screening for AD (84).

However, there is instability in different studies, which leads to doubts about the high diagnostic accuracy of the P-tau marker and the nearly equal diagnostic accuracy of plasma P-tau and cerebrospinal fluid P-tau. Based on these issues, many research teams have also proposed relevant views (85). On the one hand, it is necessary to verify the specificity and sensitivity of P-tau217, P-tau231 or P-tau181 in different cohorts under the same conditions, because different preconditioning mechanisms, detection methods and antibodies may cause differences in results (86). On the other hand, intracellular P-tau217 levels are lower than their extracellular levels in the central nervous system, suggesting that certain P-tau isomers are selectively released. P-tau217 induces tau hyperphosphorylation at multiple other sites, exacerbates tau fibrosis and cognitive impairment, and is associated with Aβ, while significantly increasing in AD. In addition to confirming the specificity and sensitivity of P-tau217, P-tau231, and P-tau181 in clinical cohorts, the mechanisms behind the high accuracy of these biomarkers, as well as the exact amount and overlap between tau proteins, need to be explored (87). The findings suggest that the high accuracy of plasma P-tau has important clinical and translational value. Tau is mainly produced in nerve cells in the brain, and plasma p-tau may reflect neurodegeneration and loss of blood–brain barrier integrity during disease progression, which may be one of the reasons why plasma P-tau has higher diagnostic accuracy for AD than commonly produced Aβ. At the same time, the structural characteristics of the truncated pattern of tau in plasma and cerebrospinal fluid are similar, which is an advantage of tau as a plasma biomarker for AD.

#### 4.1.3 Neurofilament light chain, NFL (NFL)

Neurofilament light chain (NfL) serves as a biomarker indicative of axonal degeneration and exhibits high sensitivity in both cerebrospinal fluid and plasma. Notably, alterations in NfL levels can be detected prior to the manifestation of clinical neurodegenerative symptoms, and these levels are elevated in various neurodegenerative disorders, including AD, amyotrophic lateral sclerosis, spinal muscular atrophy, multiple sclerosis, and Parkinson’s disease (88). NfL concentrations differ across the various stages of AD, rendering it a valuable tool for monitoring disease progression. In contrast to cerebrospinal fluid tests, which are invasive, and PET, which is costly and involves radiation exposure, blood biomarker testing offers the advantages of being non-invasive and less burdensome for patients, thus enhancing patient acceptability. The simplicity and convenience of blood sample collection enable mass screening, facilitating the early detection of individuals at high risk for AD, which is crucial for early diagnosis and prevention (89). Furthermore, as AD progresses, fluctuations in blood biomarker levels occur; for instance, NfL levels vary at different stages of the disease and can be employed to track its course. Regular testing of these biomarkers can provide insights into the progression of AD, as certain biomarkers may increase in concentration as the disease advances (90).

#### 4.1.4 Synapse-associated protein 25 (SNAP25)

Synapses are the basic structure of learning and memory, and loss of synapses is associated with cognitive decline. Some biomarkers of synaptic dysfunction have been linked to AD. Dendritic protein neurogranin (Ng), a postsynaptic protein associated with protein kinase c, is found primarily in hippocampal and cortical neurons and can bind to calmodulin and regulate long-term enhancement (91). Ng is a promising biomarker for AD with high sensitivity and specificity, and has been associated with Alzheimer’s disease-specific neurodegeneration and synaptic dysfunction (92).

In AD, SNAP25 is involved in vesicle fusion and exocytosis, and its levels in cerebrospinal fluid (CSF) are increased (93). SNAP25 1–40 can be used in the diagnosis of AD (AD vs. control group, area under the curve (AUC) 0.93) as well as differential diagnosis (AD vs. other dementias, AUC: 0.92) (94). Although the performance of direct detection of synaptic biomarkers in plasma is not ideal, these biomarkers in neuron-derived exosomes (NDEs) in plasma perform well (95).

Relatively little research has been done on their use as blood biomarkers in early diagnosis. However, given its performance in CSF and the potential of plasma NDEs, this biomarker is expected to be a powerful tool for early diagnosis of AD if interfering factors in plasma detection can be further addressed. In terms of disease monitoring, since it is related to the pathophysiological process of AD, it may be helpful to establish the relationship between changes in blood levels and disease progression to monitor the development of AD (96, 97).

#### 4.1.5 Limitations and challenges of blood-based biomarkers

Despite significant progress in the development of blood-based biomarkers for Alzheimer’s disease (AD), several challenges persist. The pre-analytical and analytical variability in measuring biomarkers such as Aβ42/40 and p-tau across different platforms and cohorts necessitates standardization. Additionally, the specificity of certain biomarkers, particularly Aβ, for AD in comparison to other amyloidopathies requires careful interpretation within the clinical context (98). Furthermore, the implementation of these tests in primary care settings demands validation across diverse populations and the establishment of clear cut-off values.

### 4.2 Ocular biomarkers

#### 4.2.1 Retinal imaging test

Although a number of biological markers for imaging, cognitive scales, cerebrospinal fluid and blood tests have been proposed for the early detection of AD, their use in the early and preclinical stages of the disease has been limited due to their low sensitivity and specificity. Studies have found that changes in the function, structure, metabolism, and blood vessels of the retina occur in the early stages of AD. The retina provides a unique “access” window to pathological changes in the brain, and current and developing ophthalmic technology offers us the possibility of detecting and characterizing subtle disease-related changes (99, 100). Due to the clear optical properties of the eye, the retina is the only place where neurons and blood vessels can be directly observed. Anatomically and developmentally, the retina is an extension of the central nervous system, consisting of multiple neurons including retinal ganglion cells (RGC), whose axons are connected to the lateral geniculate nucleus (LGN) and superior colliculus (SC), which in turn project axons onto the visual cortex (101). In addition to this, the retina is protected by the blood-retinal barrier (BRB), enabling selective blood-retinal permeability similar to the blood–brain barrier (BBB). The eye also exhibits a similar immune response process to that found in the brain and spinal cord (102). As a result, the retina provides a “strategic glimpse into the brain,” and retinal examination can be a novel, noninvasive, inexpensive, and easy-to-perform means of diagnosing AD. Various retinal changes in AD have been studied over the past three decades, propelling the field forward.

By staining with A variety of APP and Aβ-specific antibodies in the upper and medial regions of the human retina, a number of studies by 10 research teams including the Vic Deakin University School of Medicine in Australia have shown amyloid deposition and increased reactivity of phosphorylated tau proteins in the retinas of AD patients. Other studies emphasized that although intracellular APP positive was detected in the retinal tissue of AD patients after death, there was no significant Aβ plaque formation (103, 104). The accumulation of Aβ and the accumulation of tiny deposits in the retinal ganglion cell layer (GCL) and NFL in APP/PS1 and 3xTg-AD mice as early as 2–3 months of age, several months before significant Aβ deposits are known to occur in the hippocampus and brain tissue (105, 106).

Tau protein is expressed in axon-connection-rich layers within the retina, such as the inner plexus layer (IPL) and outer plexus layer (OPL) (107). In AD, increased levels of tau phosphorylation were observed in these layers compared to normal retinal tissue (108). In animal models of AD with 3xTg and APP/PS1, in addition to microglial activation, retinal ganglion cell (RGC) loss, and impaired retinal function, staining of pTau and tau pair spiral filaments has been reported (109). In addition, the researchers found enhanced pTau immune reactivity in the inner retina in postmortem tissue of human AD, which was co-labeled with the RGC marker TUJ1 (50 kDa neuron-specific *β* 3 tubulin), further suggesting that these cells are significantly affected during the disease process (110). In addition, the phosphorylation of Aβ and tau proteins may be due to molecular pathologic changes caused by age or other retinal diseases, such as glaucoma. Further research on different neurodegenerative diseases will improve the accuracy and specificity of detecting AD using retinal amyloid deposition and tau phosphorylation (111).

In addition, imaging techniques such as optical coherence tomography (OCT), electroretinography (ERG), functional near infrared spectroscopy (fNIRS), magnetoencephalography (MEG), and transcranial magnetic stimulation (TMS) combined with eye examination may help improve the accuracy of AD risk assessment, detection, and monitoring, but related research is still in the exploratory phase. For example, OCT can be used to detect changes in the retinal nerve fiber layer (RNFL) and macular area. In patients with Alzheimer’s disease, the RNFL may be thinner, and thickness changes may also be seen in ganglion cell layer (GCL), internal plexiform layer (IPL) in macular area (112). Studies have shown that OCT observed significant thinning of RNFL in the nasotemporal and upper and lower regions in AD patients, along with thinning of the inner and outer macular rings (113). These changes have been associated with some decline in cognitive function; Delayed latency and reduced amplitude in pattern electroretinogram (pERG) were observed in AD patients and correlated with changes in RNFL thickness, which can reflect retinal ganglion cells and their function (114). In addition to studies of these imaging techniques, retinal curcumin staining has also shown some sensitivity and specificity. Curcumin is A natural fluorescent polyphenol that has an affinity for Aβ aggregates and is able to cross the blood–brain barrier and blood-retina barrier. Fluorescence imaging of curcumin in the retina after oral administration can be used to monitor pathological changes in AD (115). For example, Aβ deposition was detected at 2.5 months of age in the APP/PS1 mouse model, and A correspondence between curcumin fluorescence and Aβ deposition has also been observed in postmortem samples and *in vivo* imaging of human AD patients (116, 117).

However, the staining effect of curcumin in the retina varies from study to study, and the sensitivity and specificity of its clinical application still need to be further verified. In summary, retinal examination may contribute to the early diagnosis of AD, and has certain value for the screening, diagnosis and intervention treatment of AD.

#### 4.2.2 Intraocular fluid biomarkers

##### 4.2.2.1 Aqueous humor markers

The application of aqueous humor biomarkers in the study of AD has garnered increasing attention in recent years. Aqueous humor, a transparent liquid found in the anterior chamber of the eye, reflects the state of systemic and neurological diseases through its composition. Detectable markers in aqueous humor include specific proteins and molecules associated with AD. For instance, the accumulation of Aβ, particularly Aβ40 and Aβ42, represents a core pathological feature of AD (118). Measuring Aβ levels in aqueous humor provides insight into systemic amyloid pathology, while levels of tau protein and its p-Tau may indicate neuronal damage or the presence of neurofibrillary tangles. Additionally, cytokines such as IL-6 and interleukin-10 (IL-10), along with neuroinflammatory markers like chemokines, may signify the presence of neuroinflammation. Variations in the levels of these markers can be utilized to monitor the progression of Alzheimer’s disease and evaluate drug efficacy. Furthermore, concurrent monitoring of eye diseases, such as glaucoma, can yield more comprehensive information regarding patient pathology (119). Compared to CSF, the collection of aqueous humor is simpler and less invasive, making it suitable for early diagnosis and ongoing disease monitoring. However, variations in detection techniques and experimental conditions may result in inconsistent findings, and certain markers may lack specificity, potentially overlapping with other diseases and impacting diagnostic accuracy. The investigation of aqueous humor markers offers a promising avenue for non-invasive diagnosis and monitoring of Alzheimer’s disease; however, further exploration and validation of its clinical application are necessary.

##### 4.2.2.2 Vitreous markers

Vitreous biomarkers have been studied as potential diagnostic tools for AD in recent years. A new study from Boston Medical Center (BMC) has found that biomarkers in the vitreous fluids of the eye are associated with pathologically proven Alzheimer’s disease (AD) and markers in postmortem brain and eye tissue in cases of Chronic Traumatic Encephalopathy (CTE). This exploratory study, published in the Journal of Alzheimer’s Disease, suggests that biomarkers in vitreous fluids could serve as a surrogate for neuropathological disease (120). For example: Abnormal levels of Aβ and Tau proteins strongly correlate with disease stage; and elevated markers of inflammation that may reflect increased disease activity. Because vitreous marker testing is less invasive, samples may be taken during eye surgery such as vitrectomy (121). In addition, more recent studies have identified pathological changes in vitreous protein abnormalities. Aβ in the vitreous may pathologically link to amyloid in the brain via the retinal blood vessel barrier, and changes in levels correlate with the severity of Alzheimer’s disease. Elevated levels of Tau and p-Tau suggest neurofibrillary tangles and retinal ganglion cell damage (122). Elevated inflammatory factors, such as increased levels of IL-1β, IL-6, TNF-*α* and other cytokines, imply that the retina and vitreous body may be involved in systemic neuroinflammation. Elevated levels of oxidative stress-related markers, such as malondialdehyde and glutathione, may be associated with retinal ganglion cell damage and metabolic disorders in the vitreous. Abnormal vitreous markers are associated with retinal vascular barrier dysfunction, which may be related to retinal blood flow changes and optic neurodegeneration in Alzheimer’s disease patients (123).

Due to the lack of specificity of vitreous markers, many vitreous markers are also associated with other eye diseases or systemic diseases, which may affect the accuracy of diagnosis. Therefore, in future studies, more sensitive detection techniques can be developed to reduce the difficulty of collecting vitreous markers. At the same time, the correlation between vitreous markers and cerebrospinal fluid, aqueous humor and blood markers should be explored to build a multimodal diagnostic system (124).

##### 4.2.2.3 Tear markers

To investigate new tear biomarkers that may be useful for the diagnosis and monitoring of AD progression, a research team from South Korea used tear samples from the Discovery cohort to perform high-resolution and comprehensive proteomic analyses (125). The discovery cohort consisted of tear samples from seven healthy controls (HC), seven mild cognitive impairment (MCI), and seven AD participants. Through in-depth proteomic analysis, the authors identified 75 differentially expressed proteins (DEPs) in tears from patients with MCI and AD compared to HC, and ultimately selected the CAP1 protein. The relative protein expression of CAP1 in the tears of patients with MCI and AD was significantly changed compared to HC. Although the expression level of CAP1 did not show the most pronounced change compared to other proteins, it showed a recognizable and consistent trend of incremental expression from HC individuals to MCI patients and subsequently to AD patients (126). This clear pattern of increase during disease progression is essential for early detection, not just high expression at specific disease stages. The gradual increase in CAP1 levels underscores its potential utility as a diagnostic marker, particularly for biosensing platforms designed to identify early disease (127).

The research team also proposed a diagnostic system for AD that utilizes surface-functionalized nanomaterials (Ab-MNPs and Ab-PNPs) for capture, magnetic separation, and selective fluorescence signal amplification. The method is capable of highly sensitive and selective detection of protein biomarkers in human tears. Elevated concentrations of Aβ and p-Tau in tears may indicate A transition from MCI to AD. Other metabolic markers, such as oxidative stress-related products, can be used to monitor disease progression (128, 129).

The acquisition of tear samples is simple and safe, without complex equipment and technology, and is suitable for large-scale screening and long-term follow-up studies. In the latest report, AD patients are often associated with thinning of the retinal ganglion cell layer, and changes in the levels of inflammation and protein markers in tears may be associated with retinopathy. In addition, there is a higher incidence of dry eye in Alzheimer’s patients, which may be related to lacrimal gland dysfunction or neuropathy (130).

The study of tear markers provides a new direction for non-invasive diagnosis of Alzheimer’s disease, but more basic and clinical studies are needed to further validate its clinical practicality and accuracy.

#### 4.2.3 Limitations and challenges of ocular biomarkers

While ocular biomarkers present a promising non-invasive approach to understanding Alzheimer’s disease (AD) pathology, several limitations must be acknowledged. The specificity of retinal Aβ and p-tau signals can be confounded by common age-related ocular conditions, such as glaucoma and age-related macular degeneration. Additionally, technical variations in imaging protocols and analytical methods across studies hinder the establishment of universal diagnostic thresholds. Although the invasiveness of acquiring aqueous and vitreous humor is less than that of cerebrospinal fluid (CSF), it still restricts the scalability of these methods for population screening. Therefore, large-scale, longitudinal studies are essential to validate the diagnostic and prognostic value of ocular biomarkers and to standardize measurement techniques.

### 4.3 Other novel diagnostic methods

#### 4.3.1 Urine biomarkers

In recent years, domestic and foreign studies have found that the selectivity of AD markers in urine is increased, and the sensitivity and specificity of the diagnosis of AD are high. Among them, urine AD7c-NTP is similar to cerebrospinal fluid in diagnostic value (131). Compared with cerebrospinal fluid, urine has the advantages of non-invasive, convenient sampling, economic security, etc., and is suitable for early AD population screening. AD7c - NTP, a neuronal transmembrane phosphoprotein, is a member of the neurofilament protein family.

AD7c-NTP may be regulated by insulin or IGF-1 stimulation. There is a high density of insulin and IGF-1 receptors in brain neurons, and the impairment of insulin/IGF-1 signaling may lead to the overexpression of AD7c-NTP in these neurons, thereby accelerating neuronal degeneration and necrosis (132). Numerous studies have confirmed that AD7c-NTP can be detected in cortical neurons, brain tissue extracts, cerebrospinal fluid, and urine during the early stages of AD (133). Furthermore, Chen et al. demonstrated that the sensitivity and specificity of AD7c-NTP in urine are comparable to those in cerebrospinal fluid for diagnosing AD. They also found that the serum levels of AD7c-NTP in patients with mild cognitive impairment (MCI) [(499 ± 139) ng/L] were significantly higher than those in the healthy control group [(271 ± 105) ng/L]. However, the diagnostic sensitivity and specificity of AD7c-NTP in MCI patients were inferior to those observed in urine and cerebrospinal fluid. Currently, there are few studies on serum AD7c-NTP, and its diagnostic value remains unclear, necessitating further investigation (134). Urine is easy to collect, non-invasive, and cost-effective, making AD7c-NTP in urine a promising marker for screening AD in the future. However, the limitations of this index include the stringent requirements for urine specimens, which generally need to be collected as mid-morning samples.

#### 4.3.2 Exhaled air analysis

Professor Shen’s team has, for the first time, demonstrated that the detection of Volatile Organic Compounds (VOCs) in human exhaled air can facilitate the early identification of patients with cognitive impairment, specifically AD. This finding is anticipated to offer a more objective and straightforward method for screening cognitive impairment in the elderly population. The team recruited 1,467 community-dwelling individuals aged over 65 to undergo cognitive assessments and exhaled air collection. The VOC components in exhaled breath were analyzed using the HHPPI-TOFMS method, revealing significant differences between the cognitively impaired group and the cognitively normal group, with up to 66 distinct VOC components identified. Further ROC analysis indicated that the combined efficiency of 10 VOC components in recognizing cognitive impairment reached as high as 0.876. These components included benzaldehyde, ethylene glycol monoethyl ether, isoallyl acetate, butadiene, toluene, ionized products of butadiene, acrolein, cyclohexane, methyl propionate, and methyl mercaptan. This innovative approach is non-invasive, objective, and cost-effective, offering new avenues for the early identification of cognitive impairment (135, 136). Additionally, given that Mild Cognitive Impairment (MCI) and dementia represent two stages of the same disease, there is currently a lack of effective biomarkers to differentiate between them. Five VOCs were found to be significantly different between the MCI group and the dementia group, namely ethylene glycol monoethyl ether, isoallyl acetate, toluene, cyclohexane, and methyl propionate, with a combined efficacy of up to 0.727 in recognizing mild cognitive impairment (137, 138). To further elucidate whether the identified characteristic VOC components reflect neurodegenerative changes, the study also measured the levels of neurofilament light chain (NfL) in the peripheral blood of elderly participants. Results indicated that NfL levels were significantly elevated in patients with cognitive impairment. The combination of the 10 identified VOC components with NfL levels can further enhance the recognition efficiency of cognitive impairment. Moreover, three VOC components (benzaldehyde, isopropenyl acetate, and toluene) were found to have a significant positive correlation with NfL levels, suggesting that VOCs may reflect neurodegenerative changes to some extent (139).

Therefore, the combination of VOCs components in human exhaled breath can more accurately identify patients with cognitive impairment in the community population, and effectively distinguish between mild cognitive impairment and dementia patients. This simple and objective method is expected to be used for large-scale screening of cognitive impairment in elderly people, and ultimately achieve early diagnosis and treatment of senile dementia.

#### 4.3.3 Skin biopsy

In one study, fibroblasts were cultured from a patient’s skin sample and imaged, measuring the total amount of cells gathered and the area of cells gathered, and reading out their changes at different time points. The study showed that Alzheimer’s patients formed fewer large clumps than non-patients, and that Alzheimer’s cells began to clump together and sink into themselves. This morphologic test shows 100% sensitivity and specificity compared to autopsy diagnosis (140).

After morphological imaging, this result is also known as the “anchor” of the test, and the first confirmative diagnosis is the protein kinase C epsilon biomarker, which is a “signature driver of synaptic change.” Synaptic loss is strongly associated with the development of AD, but this protein degrades rapidly in the patient’s blood (141).

In contrast, it is much more stable in the skin, which has led developers to use skin punch biopsy samples. Fibroblasts grown from the skin samples were exposed to a toxic oligomer that was detected by ELISA for changes in PKC epsilon, and when treated with the oligomer, PKC epsilon levels were upregulated in Alzheimer’s patients and downregulated in non-Alzheimer’s patients. When validated against a postmortem diagnosis, the test had 100% sensitivity and 96% specificity (142).

If imaging and the PKC epsilon test do not agree, an alternative proteomic biomarker and a second confirmatory test are employed to measure the levels of phosphorylated ERK-1 and ERK-2 proteins. The properties of these proteins vary among different cell types, influenced by the patient’s health status—whether they are healthy, have AD, or suffer from non-Alzheimer’s dementia (143). In this assay, fibroblasts are exposed to an inflammatory agonist, which reveals alterations in the quantities of phosphorylated ERK-1 and ERK-2. These levels are quantified using a Western blot assay, with an index developed to differentiate between Alzheimer’s and non-Alzheimer’s conditions (144). A study published in 2006 in PNAS validated the postmortem diagnosis, reporting a sensitivity of 97% and specificity of 94% for the test. A combination of results from these tests is utilized to ascertain whether a patient has Alzheimer’s disease or non-Alzheimer’s dementia, along with a detailed breakdown of each test outcome. The entire process requires approximately eight to ten weeks, with six weeks allocated for cell culture. Traditional screening methods for Alzheimer’s typically involve PET imaging and cerebrospinal fluid-based tests, which can take up to twelve weeks in total. In addition to utilizing skin samples, Amato noted that the primary distinction between Discern and other Alzheimer’s tests lies in the fact that its biomarkers have been validated postmortem and clinically confirmed as indicative of AD (145).

#### 4.3.4 Limitations and challenges of other novel diagnostic methods

Novel methods such as urine AD7c-NTP, exhaled VOCs, and skin biopsy present unique opportunities but also face significant hurdles. Urine AD7c-NTP requires strict sample collection protocols, and its levels can be influenced by renal function. The diagnostic specificity of exhaled VOCs for Alzheimer’s disease (AD) versus other respiratory or metabolic conditions requires further validation in larger cohorts. Although skin biopsy demonstrates high sensitivity and specificity in research settings, it is invasive, time-consuming (taking weeks for cell culture), and lacks standardization and widespread clinical validation. Additionally, the high cost and technical expertise required for some of these methods may limit their broader application.

## 5 Trends in diagnostic technology

### 5.1 Research diagnosis of multimodal diagnosis

AD is a progressive disease with different pathophysiological changes at different stages. Combining multiple in-vitro diagnostic methods can better track the progression of the disease. In the early stage of the disease, it is mainly manifested by abnormal deposition of Aβ, which can be detected by measuring the ratio of Aβ42/Aβ40 in the cerebrospinal fluid (146). As the disease progresses, tau protein becomes hyperphosphorylated and accumulates within neurons, leading to neuronal damage and death. At this point, measuring the levels of total tau (t-tau) and phosphorylated tau (p-tau) in cerebrospinal fluid can reflect the degree of disease progression (147). The effectiveness of AD treatment regimens varies from individual to individual. Multimodal *in vitro* diagnostics can help assess treatment response. For example, for patients who are being treated with anti-Aβ drugs, in addition to observing improvement in clinical symptoms, it is also possible to assess whether the drugs are effective in reducing Aβ deposits by detecting changes in Aβ levels in the cerebrospinal fluid. At the same time, looking at relevant biomarkers in the blood and the results of neuroimaging tests, such as changes in the degree of brain atrophy, can give a more complete picture of the impact of treatment on the patient’s overall condition. This provides a basis for personalized adjustment of the treatment regimen (148).

#### 5.1.1 Mining and combined application of novel biomarkers

The discovery of novel biomarkers has been significantly advanced through the mining and integrated application of innovative techniques, including combination and omics approaches for blood biomarkers. Researchers are not only concentrating on the aforementioned blood biomarkers but are also investigating additional blood markers for the diagnosis of Alzheimer’s disease (AD). Concurrently, they are exploring new markers associated with AD using methodologies such as genomics, proteomics, and metabolomics. Recent studies have indicated that exosomes in plasma are abundant in proteins, nucleic acids, and other biomolecules, suggesting that the miRNA expression profiles in plasma exosomes of AD patients differ from those of healthy individuals. These miRNAs may play a role in the pathological processes of AD, including the regulation of Aβ production and tau protein phosphorylation. The combined detection of miRNA in exosomes alongside traditional blood markers (such as Aβ and tau protein-related fragments) is anticipated to enhance the accuracy of early AD diagnosis (149, 150).

In genomics, genome-wide association studies (GWAS) have identified multiple gene loci associated with AD, and certain mutations or polymorphisms of these genes may be associated with the risk of developing AD. In proteomic studies, some proteins that are differentially expressed in the brain and cerebrospinal fluid of AD patients, such as complement protein C3, etc., have been found, which may be related to the neuroinflammatory process of AD. At the same time, metabolomic studies have found altered levels of certain metabolites, such as sphingolipids, in the cerebrospinal fluid and blood of AD patients. These findings provide a rich resource for the multimodal diagnosis of AD by combining multiple markers (151).

#### 5.1.2 A diagnostic platform integrating multiple technologies

Microfluidic chip technology enables the integration of multiple steps, including sample processing and biomarker detection, on a compact chip. For instance, microfluidic chips designed to detect both Aβ and tau proteins in cerebrospinal fluid are currently under development (152). These chips can process a limited number of samples rapidly and efficiently, facilitating the simultaneous detection of multiple biomarkers by incorporating various detection techniques, such as immunoassays and electrochemical detection. This innovation positions microfluidic chips as vital tools for the *in vitro* diagnosis of AD in the future (153). Moreover, novel biosensors are emerging that can detect AD-related biomarkers. For example, biosensors utilizing nanomaterials, such as graphene, exhibit high sensitivity in detecting Aβ oligomers in blood. Additionally, these sensors can be integrated to simultaneously detect different markers, such as combining a sensor for Aβ with one for tau protein, thereby enabling multimodal diagnostics. They can also be coupled with other technologies, such as microfluidic systems, to create more complex and efficient diagnostic platforms (154).

#### 5.1.3 The application prospect of multimodal diagnosis in clinic

With the advancement of multimodal diagnostic technology, relatively simple and convenient combined detection methods are anticipated to be promoted within community and primary healthcare settings. For instance, by integrating several blood biomarkers with a straightforward cognitive assessment tool, individuals at high risk for AD can be identified at an early stage. For these individuals, proactive lifestyle interventions (such as increased physical activity and dietary control) and pharmacological interventions (including health products or medications aimed at enhancing cognitive function) can be implemented to delay the onset of AD (155). A risk prediction model for AD can be established using multimodal diagnostic data in conjunction with the patient’s family history and lifestyle factors. By screening large populations, it becomes feasible to accurately predict an individual’s risk of developing AD. For example, in individuals with a familial history of the disease, a combination of genetic marker testing, blood biomarker analysis, and regular cognitive assessments can forecast the likelihood of developing AD decades in advance, thereby providing guidance for long-term health management (156). Based on the outcomes of multimodal diagnoses, healthcare professionals can select the most appropriate treatment for patients. For instance, patients with Aβ deposition may be preferentially treated with monoclonal antibody therapies targeting Aβ, while those with concurrent neuroinflammation may require a combination of anti-inflammatory medications in addition to anti-Aβ therapies. This precise treatment selection aims to enhance treatment efficacy and minimize adverse drug reactions. In clinical trials for new AD drugs, multimodal diagnosis can facilitate more accurate subject screening and drug efficacy evaluation. For example, patients at specific disease stages can be selected for clinical trials based on detailed biomarker detection and cognitive function assessments. The effects of the drugs on biomarkers and clinical symptoms can be continuously monitored through multimodal diagnostic approaches during the trial, allowing for a more objective evaluation of therapeutic effects and accelerating the development of AD therapeutics (157).

### 5.2 Biosensors and nanotechnology

With the accelerating aging of the global population, AD has become a major medical challenge that cannot be ignored. The disease not only relentlessly erodes patients’ memory and thinkingskills, but also dramatically affects the quality of their daily lives, along with the need for long-term care. Therefore, early detection and accurate assessment of the progression of AD is of immeasurable value to patients and their families. However, the road to diagnosis of chronic neurodegenerative diseases such as AD is not easy and often comes with high healthcare costs, especially in the early stages of the disease, when cognitive decline has just begun. To overcome these obstacles, researchers in recent years have focused on exploring a variety of promising biomarkers and behavioral characteristics to enable earlier and more accurate diagnosis of Alzheimer’s disease. At the same time, they have developed cutting-edge biosensor pieces and nanotechnology that are “tailored” to different markers and physiological problems, which demonstrate great potential in Alzheimer’s detection.

#### 5.2.1 Application potential of biosensors in *in vitro* diagnosis of AD

As a progressive neurodegenerative disease, current clinical diagnostic techniques for AD are often costly, time-consuming, and invasive, significantly limiting in-depth research on AD-specific markers and the innovation and development of efficient devices required for point-of-care testing (POCT) (158). To address this challenge, the scientific community is actively exploring and evaluating a variety of biosensing technologies aimed at overcoming existing bottlenecks. Professor Mohamad Sawan led a research team to analyze biomarkers and biosensing technologies closely related to AD, summarizing the latest research results in the field of early detection technology. The team systematically investigated potential Alzheimer’s biomarkers identified in various body fluids and behavioral patterns in recent years. They further explored the underlying mechanisms of different biosensing technologies, as well as the dilemmas and challenges faced in diagnosing the disease. On this basis, we particularly highlight the development potential of novel biosensors designed to capture the diverse characteristics of diseases and provide robust technical support for early diagnosis in the POCT field (159). Biosensors are capable of translating changes in Alzheimer’s biomarkers into measurable signals through sensitive conversion mechanisms. Biomarkers related to Alzheimer’s disease have been identified in various body fluids, including blood, cerebrospinal fluid, saliva, tears, and sweat. For instance, markers such as Aβ42/40 and p-tau217 can be detected in blood, while Aβ42 and t-tau are detectable in cerebrospinal fluid (160). With their specific recognition elements and signal conversion mechanisms, biosensors can detect these markers with high sensitivity. For example, electrode modification techniques in electrochemical sensors (such as modifying electrodes with nanomaterials) can enhance the detection sensitivity of biomarkers. A glass carbon electrode modified with SnO2 nanofibers has a detection limit of up to 0.638 fg/mL for Aβ42 (161). Some electrochemical sensors determine biomarker concentrations by detecting changes in current generated by REDOX reactions, effectively detecting low concentrations of biomarkers in early-stage patients. Additionally, some biosensors can monitor dynamic changes in biomarker concentrations in real-time (162). Optical sensors can utilize fluorescence or surface plasmonic resonance (SPR) signal changes to monitor the concentrations of Aβ and tau proteins in real-time during the treatment of Alzheimer’s disease patients, thereby providing a basis for evaluating treatment effects. In drug clinical trials, these sensors can continuously observe the effects of drugs on biomarker levels, assisting in the adjustment of treatment regimens (163).

At the same time, biosensor technology also faces a number of challenges. On the one hand, the performance of biosensors can be affected by environmental factors. The enzyme activity in enzyme-based biosensors changes due to changes in temperature and humidity, resulting in unstable detection results. In the course of multiple uses, the biometric elements on the surface of the sensor are prone to denaturation or fall off, which will affect the repeatability of the detection. On the other hand, the composition of human samples is complex, and substances such as proteins and lipids in blood and cerebrospinal fluid will be adsorbed on the surface of the sensor non-specifically, interfering with the specific binding of biomarkers and sensors, resulting in false positive or false negative results. In addition, the manufacturing process of some high-precision biosensors is complex and the materials are expensive, such as the MEMS -based biosensors, which have high manufacturing equipment and process costs, which also limits their large-scale clinical application (164). Based on the many challenges faced, the latest research team has proposed some future directions. For example, multifunctional biosensors that integrate multiple detection principles can be developed, while electrochemical and optical detection methods can be used to improve the accuracy and reliability of detection. Combining MEMS and artificial intelligence technology, intelligent micro-biosensors can be built to automate sample processing, detection and data analysis, and transmit data through wireless communication. In addition, it can also be deeply integrated with other technologies, such a nanotechnology and microfluidic technology. Nanomaterials can be used as signal amplification tags or for enrichment biomarkers, thus improving detection sensitivity; Microfluidic technology can build a miniaturized *in vitro* diagnostic platform to realize rapid sample detection and automated operation (165).

#### 5.2.2 Application potential of nanotechnology in in vitro diagnosis of AD

Aβ plaques and harmful inflammation are the two primary symptoms of AD. However, due to the absence of dual-target therapeutic functions, BBB penetration, and low imaging sensitivity, precise treatment options for AD are currently unavailable. In response to this challenge, researchers have collaborated to develop a near-infrared Region II aggregation-induced luminescence (AIE) nanodiagnostic system aimed at the precise treatment of AD. At a wavelength of 1,350 nm, the anti-quench luminescence effectively monitors BBB penetration *in vivo* and the specific binding of the nanotherapeutic system to plaques. Triggered by reactive oxygen species (ROS), two encapsulated therapeutic AIE molecules are released in a controlled manner to activate a self-reinforcing therapeutic program. One of these molecules specifically inhibits the formation of Aβ fibers, degrades existing Aβ fibers, and prevents reaggregation through multiple competitive interactions. This process has been validated through computational analysis, further alleviating inflammation. The second molecule effectively clears ROS and inflammation, restores the brain’s REDOX balance, enhances the therapeutic effect, and jointly reverses neurotoxicity, resulting in significant behavioral and cognitive improvements in a female AD mouse model (166). In this study, two therapeutic aggregation-induced luminescence (AIE) molecules (AIEgens) with near-infrared-II (NIR-II) emission, specifically Compound 3 and Compound 6, were synthesized. Compound 3 demonstrated the ability to specifically inhibit the formation of Aβ fibers and decompose Aβ plaques through van der Waals forces, hydrogen bonding, and *π*-π interactions. Its binding affinity for Aβ fibers is comparable to that of conventional thioflavin T (ThT), with a dissociation constant (K_d) that is closely aligned. This specific binding enhances the sensitivity of assays to accurately identify Aβ protein abnormalities associated with AD (167). Compound 6, incorporated into the nanocomposite (NCs), possesses a Ce(III) active center, which effectively clears harmful inflammation-associated ROS and indirectly enhances the sensitivity of detecting AD-related pathological features through its impact on inflammation-related factors. Additionally, the study found that NCs exhibit favorable optical properties, with absorption and emission spectral characteristics that facilitate high-sensitivity detection. For instance, NCs emit at 1350 nm, demonstrating high sensitivity at this wavelength, which effectively monitors the cross-skull signal of Aβ plaques combined with NCs *in vivo*, providing a highly sensitive diagnostic tool for *in vitro* detection of AD (168, 169). Nonetheless, several risks and challenges are associated with this innovative technology. Firstly, to achieve high-sensitivity, long-wavelength detection of Alzheimer’s using IR-II emission, the molecular structure must be meticulously balanced. For example, it is essential to ensure a high quantum yield while maintaining a substantial *π*-conjugated structure for long-wavelength absorption (170). Furthermore, while ensuring a strong Aβ-affinity, the molecular structure should not become overly complex, as this could adversely affect other properties, thereby imposing significant demands on molecular design. Secondly, the ideal nanomaterials for *in vitro* diagnosis of Alzheimer’s disease must possess multiple interaction sites and strong Aβ-affinity to effectively inhibit Aβ fiber generation, degradation, and other multi-target functions. However, designing such molecular structures presents challenges, as the synergies of multiple interactions must be carefully considered, including a rational combination of van der Waals forces, *π*-π stacking, hydrogen bonding, and other interactions.

Finally, although nanomaterials have shown good detection performance in *in vitro* experiments, there may be biocompatibility and safety issues when applied *in vivo*, among others. For example, nanomaterials may cause immune reactions and cytotoxicity, which limits their further application in clinical in vitro diagnosis (171).

In summary, the future development of nanotechnology has the following prospects: First, nanomaterials can be designed to integrate multiple diagnostic functions, such as combining imaging functions with the detection of disease markers and the evaluation of disease progression. It is an important direction to develop nanomaterials that can not only detect Aβ protein with high sensitivity, but also monitor A variety of biomarkers related to Alzheimer’s disease, such as inflammation-related factors and REDOX status. Second, the intelligent responsiveness of nanomaterials should be further optimized to better adapt to the complex pathological environment of Alzheimer’s disease. For example, nanomaterials that are responsive to multiple biomarkers or pathological signals can be designed so that precise diagnosis can be made according to different stages of the disease or the pathological characteristics of individuals. Third, in the process of the transformation of nanotechnology into clinical *in vitro* diagnosis, it is necessary to strengthen the safety assessment of nanomaterials, including comprehensive biocompatibility studies and long-term toxicity studies, to ensure their safety in clinical applications.

### 5.3 AI-assisted diagnosis

With the development of artificial intelligence technology, more and more research is exploring how to use AI technologies such as deep learning and convolutional neural networks to play a role in the prediction, diagnosis and treatment of Alzheimer’s disease quickly and efficiently, involving areas such as imaging analysis, neuropsychological data processing, treatment, as well as intelligent assistive devices. With its powerful data processing and analysis capabilities, artificial intelligence has brought new methods and perspectives to AD *in vitro* diagnosis. Through the mining and analysis of multi-source data, as well as the application of image recognition technology, AI has shown great potential in Alzheimer’s diagnosis. At the same time, its advantages and limitations also point the way for future development.

In 2018, the National Institute on Aging - Alzheimer’s Disease Association (NIA-AA) formally proposed the diagnostic framework for AD based on the ATN criteria: A- confirmed by cerebrospinal fluid or Aβ-PET; T- confirmed by phosphorylated tau protein or tau-PET in cerebrospinal fluid; N- confirmed by total tau levels in cerebrospinal fluid, FDG-PET, or MRI brain atrophy (2, 71). Artificial intelligence technologies, particularly machine learning and image processing algorithms, are instrumental in the early screening, diagnosis, and prognostic follow-up of AD through the analysis of brain scan image data. Several studies have identified texture differences between AD patients and normal controls in specific brain structures, such as the hippocampus, corpus callosum, and thalamus (52, 172). These texture differences may indicate a heterogeneous distribution of microscopic structures within these regions. Consequently, model building and machine learning methods have increasingly become the focus of radiomic research in AD. The radiomic features and classification models derived from imaging data, which is the most commonly utilized dataset in AD studies, including MRI and PET, are considered promising biomarkers for diagnosing AD and MCI. Early clinical research primarily compared AD patient groups with normal control groups, employing traditional machine learning methods such as multi-layer perceptrons and autoencoders. These methods typically achieve a classification accuracy of approximately 0.91 (173). Furthermore, enhancements in traditional machine learning approaches, including Bayesian linear regression, Gaussian process regression, and elastic net regression, have demonstrated classification accuracies of around 0.95 (174). With the advancement of deep learning, researchers Hon and Khan have integrated two prominent deep learning architectures, VGG-16 (a convolutional neural network with 16 layers designed for object detection and classification) and InceptionV4 (an image recognition algorithm model utilizing multi-scale and multi-layer network structures), for image data analysis, yielding superior performance (175).

#### 5.3.1 Analysis of image data by artificial intelligence

Artificial intelligence technology can use machine learning and image processing algorithms to analyze brain scan image data, and play an important role in the early screening, diagnosis and prognosis follow-up of AD. In MRI, sMRI can detect the abnormal changes of cerebral cortex morphology, volume and white matter, providing a means for early diagnosis, and the application of automatic brain segmentation, quantitative analysis and machine learning can help improve the diagnostic accuracy (176). fMRI can detect brain activity and regional connectivity changes, and AI can identify biomarkers through deep learning and compare the data of healthy people, thus diagnosing AD with a high accuracy (177). DTI imaging can obtain the structural changes of nerve fiber bundles, and AI processes the image data and detects the changes through machine learning algorithms for determining clinical stages (178). In PET, AI implements automatic analysis of PET images through algorithms such as machine learning and artificial neural network, mining features conducive to diagnosis from a large number of images, and improving the accurate diagnosis rate.

#### 5.3.2 Comprehensive analysis of multi-source data by artificial intelligence

AI algorithms can be applied to a variety of data sets, including MRI scans, genetic information, clinical data, and neuropsychological data. In the realm of neuropsychological data, machine learning methods are employed to analyze the acoustic, semantic, and syntactic components of speech recordings, with deep learning models demonstrating high accuracy in identifying early AD. By utilizing dimensional assessment methods that integrate artificial intelligence with virtual reality and daily activity instruments, diagnostic classification models can be developed, facilitating early diagnosis and treatment management (179). Regarding genetic data, the research team constructed a deep learning model to evaluate polygenic risk and stratify individuals, thereby providing a foundation for early screening. Furthermore, the developed UKB-DRP model combines multiple factors and exhibits high predictive power and practicality for all-cause dementia and AD (180).

At present, AI research in the field of AD faces many challenges, such as improving credibility and transparency. The reliability of AI models in forecasting and decision-making is a key issue. To improve the credibility of models, researchers need to develop models with high accuracy and stability, with adequate validation. In addition, it is important to improve the transparency and interpretability of the models, so that doctors and patients can understand the basis of the models’ decisions, thereby increasing trust in the models. Improving reproducibility is also key, which is very important in AI research, and ensuring the availability and verifiability of data sets used in studies is essential. Open sharing of data sets and methods can promote the reproducibility of studies, and also facilitate comparison and validation between different research teams to address the heterogeneity of AD clinical. The clinical characteristics of AD are heterogeneous because the condition may vary from patient to patient. This means that it is challenging to develop AI tools that are suitable for a variety of clinical situations (181). To improve the effectiveness of personalized diagnostic and predictive tools, researchers need to develop tools that can handle different imaging modes and clinical features, and systematically quantify and account for clinical heterogeneity. Ideally, these AI tools will be able to achieve the same results regardless of imaging protocols, machines used, or population changes. Improving scalability is also a key point, and products that can be widely used are good products. Despite these challenges, remarkable progress has been made in the application of AI to AD research. By analyzing multiple types of data, AI can predict Alzheimer’s disease years before symptoms appear. By analyzing a patient’s brain MRI scans, speech, and combining genetic data, AI can spot potential signs of AD and assess risk. The UKB-DRP model can predict all-cause dementia and AD 5, 10 or more years into the future, providing potential for early intervention (182).

At the same time, artificial intelligence can also reduce the workload of doctors. Faced with a large amount of image data and complex clinical information, doctors’ diagnosis work is heavy and easy to fatigue. AI can automatically process and analyze data, quickly provide diagnostic suggestions, assist doctors in decision-making, improve diagnostic efficiency, and enable doctors to devote more energy to complex cases and patient communication. The innovation of AI technology in diagnosis, prediction and treatment is expected to provide more effective tools and methods for AD management. With continued research and technological advancements, we can expect AI to play an even greater role in helping patients and clinicians deal with AD.

## 6 Discussion

### 6.1 Summary and challenges

*In vitro* diagnostic technology holds significant promise for the early diagnosis, disease staging, and treatment monitoring of Alzheimer’s disease (AD). Standardization and validation of diagnostic methods can enhance the specificity and sensitivity of biomarkers, reduce costs, increase accessibility, and ultimately improve patient outcomes and quality of life. During the early stages of AD, when patients exhibit only mild cognitive impairment, *in vitro* diagnostic technology can effectively identify disease indicators by analyzing specific biomarkers in blood, cerebrospinal fluid, and other biological samples. For instance, measuring the levels of Aβ 1–42, total tau, and phosphorylated tau in cerebrospinal fluid can facilitate early diagnosis. Recent advancements in high-throughput proteomics have furthered the investigation of peripheral blood biomarkers, such as p-tau231 in plasma, demonstrating high accuracy in distinguishing AD patients from those with non-AD neurodegenerative disorders. As AD progresses, its pathological and clinical manifestations become increasingly complex. *In vitro* diagnostics that track dynamic changes in biomarkers, in conjunction with imaging technologies, can provide accurate disease staging. This information allows healthcare providers to devise personalized treatment plans, which may include tailored drug therapies and rehabilitation training regimens, thereby advancing the concept of precision medicine. For example, in the early stages of the disease, patients may be encouraged to maintain social interactions and engage in hobbies based on their diagnostic findings. In the intermediate and late stages, appropriate levels of care can be administered. Additionally, by considering the patient’s biomarker levels, physicians can select the most suitable medications and dosages to enhance therapeutic efficacy, minimize side effects, and improve the overall quality of life for patients. Although *in vitro* diagnosis now has a very broad application prospect for the early diagnosis, disease staging and treatment monitoring of AD, in order to ensure the accuracy and reliability of diagnostic results, it is necessary to establish unified diagnostic criteria and validation methods, improve the specificity and sensitivity of biomarkers, and reduce costs and improve accessibility. These measures still need more in-depth research and exploration.

Currently, one of the primary challenges in the clinical application of Alzheimer’s disease (AD) fluid markers is the absence of standardized protocols for fluid collection, processing, storage, and detection. For instance, Aβ42 in cerebrospinal fluid (CSF) and blood is prone to adhesion and aggregation, while phosphorylated tau (p-tau) is affected by environmental factors, such as temperature, which can lead to fluctuations in marker concentrations during sample handling and detection. Consequently, establishing uniform standards for sample collection, processing, and storage is crucial to ensuring the accuracy and consistency of diagnostic results (183). Existing single biomarkers frequently encounter issues with “interfering signals” and lack specificity; for example, certain inflammation-related markers may be elevated in both AD and brain infections. To address this, integrating multidimensional biomarkers—such as indicators reflecting the abnormal metabolism of Aβ, hyperphosphorylation of tau protein, and damage to the neurovascular unit—can facilitate the creation of a dedicated AD “diagnostic fingerprint” that accurately eliminates “false positive” interference. The development of high-precision detection technologies, such as single molecule array (Simoa) technology, can significantly enhance the specificity and sensitivity of biomarker detection. Furthermore, the combination of multiple biomarkers can improve diagnostic accuracy; for instance, integrating markers such as Aβ42, p-tau, and NfL allows for a more precise diagnosis of AD (184). In the realm of early AD diagnosis, some relevant biomarkers are characterized by extremely low levels in the body.

Early screening through *in vitro* diagnostic technology can significantly reduce healthcare costs. By utilizing blood marker detection and neuropsychological assessment, community memory clinics can effectively exclude low-risk individuals and refer those at medium to high risk to cognitive centers for diagnosis. Personalized treatment approaches can enhance outcomes, minimize the waste of medical resources, and improve cost-effectiveness. However, despite the promising scientific research outcomes achieved by advanced in vitro diagnostic technologies, their clinical implementation is often hindered by prohibitive costs. For instance, the detection of AD through single-cell sequencing and high-resolution mass spectrometry incurs substantial expenses for both equipment and consumables, making it unaffordable for many medical institutions. Developing economical, modular technologies, such as microfluidic chips with integrated immunoassays, is essential for providing high-quality diagnostics at accessible prices, thereby addressing this challenge. Even with reduced costs, the adoption of these technologies remains problematic. Remote areas often lack the necessary expertise to operate complex equipment, while deficiencies in cold chain logistics can adversely affect sample inspection. Furthermore, the supporting infrastructure in primary medical institutions is often inadequate. Therefore, enhancing personnel training, addressing logistical shortcomings, and promoting the integration of hierarchical diagnosis and treatment with new technologies can enable *in vitro* diagnostics to benefit potential AD patients.

### 6.2 Regulatory and implementation considerations

The translation of novel diagnostic technologies from research to clinical practice encounters significant regulatory hurdles. Biomarker tests and devices necessitate approval from regulatory bodies such as the FDA or CE marking, a process that requires robust clinical validation (185). Cost-effectiveness and reimbursement policies are crucial for widespread adoption. Although blood-based tests are less expensive than PET or CSF analyses, their integration into healthcare systems must demonstrate long-term economic benefits. Implementation in primary care settings demands user-friendly formats, minimal training requirements, and clear interpretation guidelines to ensure accessibility and correct usage outside specialized centers (186).

Although the challenges associated with AD *in vitro* diagnosis are significant, the future appears promising. Collaborative efforts across all sectors to overcome these obstacles will undoubtedly provide a strong impetus for the prevention and treatment of AD, offering renewed hope for patient recovery.

## References

[1] Scheltens P De Strooper B Kivipelto M Cummings J van der Flier WM. Alzheimer's disease. Lancet. (2021) 397:1577–90. doi: 10.1016/s0140-6736(20)32205-4, PMID: 33667416 PMC8354300

[2] Lane CA Hardy J Schott JM. Alzheimer's disease. Eur J Neurol. (2018) 25:59–70. doi: 10.1111/ene.13439, PMID: 28872215

[3] Graff-Radford J Yong KXX Apostolova LG Bouwman FH Carrillo M Dickerson BC. New insights into atypical Alzheimer's disease in the era of biomarkers. Lancet Neurol. (2021) 20:222–34. doi: 10.1016/s1474-4422(20)30440-3, PMID: 33609479 PMC8056394

[4] Ju Y Tam KY. Pathological mechanisms and therapeutic strategies for Alzheimer's disease. Neural Regen Res. (2022) 17:543–9. doi: 10.4103/1673-5374.320970, PMID: 34380884 PMC8504384

[5] Hampel H Hu Y Cummings J Mattke S Iwatsubo T Nakamura A. Blood-based biomarkers for Alzheimer's disease: current state and future use in a transformed global healthcare landscape. Neuron. (2023) 111:2781–99. doi: 10.1016/j.neuron.2023.05.017, PMID: 37295421 PMC10720399

[6] Glenner GG Wong CW. Alzheimer's disease: initial report of the purification and characterization of a novel cerebrovascular amyloid protein. Biochem Biophys Res Commun. (1984) 120:885–90. doi: 10.1016/s0006-291x(84)80190-4, PMID: 6375662

[7] Zhang Y Song W. Islet amyloid polypeptide: another key molecule in Alzheimer's pathogenesis? Prog Neurobiol. (2017) 153:100–20. doi: 10.1016/j.pneurobio.2017.03.001, PMID: 28274676

[8] Deng Y Wang Z Wang R Zhang X Zhang S Wu Y. Amyloid-β protein (aβ) Glu11 is the major β-secretase site of β-site amyloid-β precursor protein-cleaving enzyme 1(BACE1), and shifting the cleavage site to aβ Asp1 contributes to Alzheimer pathogenesis. Eur J Neurosci. (2013) 37:1962–9. doi: 10.1111/ejn.12235, PMID: 23773065

[9] Cheignon C Tomas M Bonnefont-Rousselot D Faller P Hureau C Collin F. Oxidative stress and the amyloid beta peptide in Alzheimer's disease. Redox Biol. (2018) 14:450–64. doi: 10.1016/j.redox.2017.10.014, PMID: 29080524 PMC5680523

[10] Bitel CL Kasinathan C Kaswala RH Klein WL Frederikse PH. Amyloid-β and tau pathology of Alzheimer's disease induced by diabetes in a rabbit animal model. J Alzheimer's Dis. (2012) 32:291–305. doi: 10.3233/jad-2012-120571, PMID: 22785400

[11] Viola KL Klein WL. Amyloid β oligomers in Alzheimer's disease pathogenesis, treatment, and diagnosis. Acta Neuropathol. (2015) 129:183–206. doi: 10.1007/s00401-015-1386-3, PMID: 25604547 PMC4390393

[12] De Felice FG Vieira MN Bomfim TR Viola KL Zhao WQ Ferreira ST. Protection of synapses against Alzheimer's-linked toxins: insulin signaling prevents the pathogenic binding of Abeta oligomers. Proc Natl Acad Sci USA. (2009) 106:1971–6. doi: 10.1073/pnas.0809158106 19188609 PMC2634809

[13] Lourenco MV Clarke JR Frozza RL Bomfim TR Forny-Germano L Batista AF. TNF-α mediates PKR-dependent memory impairment and brain IRS-1 inhibition induced by Alzheimer's β-amyloid oligomers in mice and monkeys. Cell Metab. (2013) 18:831–43. doi: 10.1016/j.cmet.2013.11.002, PMID: 24315369

[14] Mair W Muntel J Tepper K Tang S Biernat J Seeley WW. FLEXITau: quantifying post-translational modifications of tau protein in vitro and in human disease. Anal Chem. (2016) 88:3704–14. doi: 10.1021/acs.analchem.5b04509, PMID: 26877193 PMC5808556

[15] Basurto-Islas G Gu JH Tung YC Liu F Iqbal K. Mechanism of tau hyperphosphorylation involving lysosomal enzyme asparagine endopeptidase in a mouse model of brain ischemia. J Alzheimer's Dis. (2018) 63:821–33. doi: 10.3233/jad-170715, PMID: 29689717

[16] Rekha A Afzal M Babu MA Menon SV Nathiya D Supriya S. GSK-3β dysregulation in aging: implications for tau pathology and Alzheimer's disease progression. Mol Cell Neurosci. (2025) 133:104005. doi: 10.1016/j.mcn.2025.104005, PMID: 40120784

[17] Mattioni A Carsetti C Bruqi K Caputo V Cianfanelli V Bacalini MG. A variant of the autophagic receptor NDP52 counteracts phospho-TAU accumulation and emerges as a protective factor for Alzheimer's disease. Cell Death Dis. (2025) 16:300. doi: 10.1038/s41419-025-07611-2, PMID: 40234443 PMC12000434

[18] Heneka MT Carson MJ El Khoury J Khoury Joseph El Landreth Gary E Brosseron Frederic. Neuroinflammation in Alzheimer's disease Lancet Neurol (2015). 14. 388–405 doi: 10.1016/s1474-4422(15)70016-5 25792098 PMC5909703

[19] Adams JN Harrison TM Maass A Baker SL Jagust WJ. Distinct factors drive the spatiotemporal progression of tau pathology in older adults. J Neurosci. (2022) 42:1352–61. doi: 10.1523/jneurosci.1601-21.2021, PMID: 34965972 PMC8883857

[20] Lee HG Lee JH Flausino LE Quintana FJ. Neuroinflammation: an astrocyte perspective. Sci Transl Med. (2023) 15: eadi7828. doi: 10.1126/scitranslmed.adi7828, PMID: 37939162

[21] Lu Y Fujioka H Wang W Zhu X. Bezafibrate confers neuroprotection in the 5xFAD mouse model of Alzheimer's disease. Biochim Biophys Acta Mol basis Dis. (2023) 1869:166841. doi: 10.1016/j.bbadis.2023.166841, PMID: 37558011 PMC10528941

[22] Song T Song X Zhu C Patrick R Skurla M Santangelo I. Mitochondrial dysfunction, oxidative stress, neuroinflammation, and metabolic alterations in the progression of Alzheimer's disease: a meta-analysis of in vivo magnetic resonance spectroscopy studies. Ageing Res Rev. (2021) 72:101503. doi: 10.1016/j.arr.2021.101503, PMID: 34751136 PMC8662951

[23] Wang C Zong S Cui X Wang X Wu S Wang L. The effects of microglia-associated neuroinflammation on Alzheimer's disease. Front Immunol. (2023) 14:1117172. doi: 10.3389/fimmu.2023.1117172, PMID: 36911732 PMC9992739

[24] Chaney AM Lopez-Picon FR Serrière S Wang R Bochicchio D Webb SD. Prodromal neuroinflammatory, cholinergic and metabolite dysfunction detected by PET and MRS in the TgF344-AD transgenic rat model of AD: a collaborative multi-modal study. Theranostics. (2021) 11:6644–67. doi: 10.7150/thno.56059, PMID: 34093845 PMC8171096

[25] Praticò D. Oxidative stress hypothesis in Alzheimer's disease: a reappraisal. Trends Pharmacol Sci. (2008) 29:609–15. doi: 10.1016/j.tips.2008.09.001, PMID: 18838179

[26] Teunissen CE Verberk IMW Thijssen EH Vermunt L Hansson O Zetterberg H. Blood-based biomarkers for Alzheimer's disease: towards clinical implementation. Lancet Neurol. (2022) 21:66–77. doi: 10.1016/s1474-4422(21)00361-6, PMID: 34838239

[27] Ashleigh T Swerdlow RH Beal MF. The role of mitochondrial dysfunction in Alzheimer's disease pathogenesis. Alzheimers Dement. (2023) 19:333–42. doi: 10.1002/alz.12683, PMID: 35522844

[28] D'Alessandro MCB Kanaan S Geller M Praticò D Daher JPL. Mitochondrial dysfunction in Alzheimer's disease. Ageing Res Rev. (2025) 107:102713. doi: 10.1016/j.arr.2025.102713, PMID: 40023293

[29] Mankhong S Kim S Lee S. Development of Alzheimer's disease biomarkers: from CSF- to blood-based biomarkers. Biomedicine. (2022) 10. doi: 10.3390/biomedicines10040850 PMC9025524 35453600

[30] Stefaniak J O'Brien J. Imaging of neuroinflammation in dementia: a review. J Neurol Neurosurg Psychiatry. (2016) 87:21–8. doi: 10.1136/jnnp-2015-311336, PMID: 26384512

[31] McDade E Cummings JL Dhadda S Swanson CJ Reyderman L Kanekiyo M. Lecanemab in patients with early Alzheimer's disease: detailed results on biomarker, cognitive, and clinical effects from the randomized and open-label extension of the phase 2 proof-of-concept study. Alzheimer's Res Ther. (2022) 14:191. doi: 10.1186/s13195-022-01124-2, PMID: 36544184 PMC9768996

[32] Shoshan-Barmatz V Nahon-Crystal E Shteinfer-Kuzmine A Gupta R. VDAC1, mitochondrial dysfunction, and Alzheimer's disease. Pharmacol Res. (2018) 131:87–101. doi: 10.1016/j.phrs.2018.03.010, PMID: 29551631

[33] Morató X Marquié M Tartari JP Lafuente A Abdelnour C Alegret M. A randomized, open-label clinical trial in mild cognitive impairment with EGb 761 examining blood markers of inflammation and oxidative stress. Sci Rep. (2023) 13:5406. doi: 10.1038/s41598-023-32515-6, PMID: 37012306 PMC10070452

[34] Chatterjee P Pedrini S Doecke JD Thota R Villemagne VL Doré V. Plasma Aβ42/40 ratio, p-tau181, GFAP, and NfL across the Alzheimer's disease continuum: a cross-sectional and longitudinal study in the AIBL cohort. Alzheimers Dement. (2023) 19:1117–34. doi: 10.1002/alz.12724, PMID: 36574591

[35] Olsson B Lautner R Andreasson U Öhrfelt A Portelius E Bjerke M. CSF and blood biomarkers for the diagnosis of Alzheimer's disease: a systematic review and meta-analysis. Lancet Neurol. (2016) 15:673–84. doi: 10.1016/s1474-4422(16)00070-3, PMID: 27068280

[36] Stoner A Fu L Nicholson L Zheng C Toyonaga T Spurrier J. Neuronal transcriptome, tau and synapse loss in Alzheimer's knock-in mice require prion protein. Alzheimer's Res Ther. (2023) 15:201. doi: 10.1186/s13195-023-01345-z, PMID: 37968719 PMC10647125

[37] Kunkle BW Grenier-Boley B Sims R Bis JC Damotte V Naj AC. Genetic meta-analysis of diagnosed Alzheimer's disease identifies new risk loci and implicates aβ, tau, immunity and lipid processing. Nat Genet. (2019) 51:414–30. doi: 10.1038/s41588-019-0358-2, PMID: 30820047 PMC6463297

[38] Manczak M Reddy PH. Abnormal interaction of VDAC1 with amyloid beta and phosphorylated tau causes mitochondrial dysfunction in Alzheimer's disease. Hum Mol Genet. (2012) 21:5131–46. doi: 10.1093/hmg/dds360, PMID: 22926141 PMC3490521

[39] Zhang H Wei W Zhao M Ma L Jiang X Pei H. Interaction between aβ and tau in the pathogenesis of Alzheimer's disease. Int J Biol Sci. (2021) 17:2181–92. doi: 10.7150/ijbs.57078, PMID: 34239348 PMC8241728

[40] Gomes LA Hipp SA Rijal Upadhaya A Balakrishnan K Ospitalieri S Koper MJ. Aβ-induced acceleration of Alzheimer-related τ-pathology spreading and its association with prion protein. Acta Neuropathol. (2019) 138:913–41. doi: 10.1007/s00401-019-02053-5, PMID: 31414210

[41] Grochowska KM Gomes GM Raman R Kaushik R Sosulina L Kaneko H. Jacob-induced transcriptional inactivation of CREB promotes aβ-induced synapse loss in Alzheimer's disease. EMBO J. (2023) 42:e112453. doi: 10.15252/embj.2022112453, PMID: 36594364 PMC9929644

[42] Cimiotti CGV Paganetti P Rossi S Soldini E Sacco L. Correlation between blood monocytes and CSF tau in Alzheimer's disease: the effect of gender and cognitive decline. NeuroSci. (2023) 4:319–30. doi: 10.3390/neurosci4040026, PMID: 39484180 PMC11523745

[43] Balusu S Horré K Thrupp N Craessaerts K Snellinx A Serneels L. MEG3 activates necroptosis in human neuron xenografts modeling Alzheimer's disease. Science. (2023) 381:1176–82. doi: 10.1126/science.abp9556, PMID: 37708272 PMC7615236

[44] Cavallaro F Conti Nibali S Cubisino SAM Caruso P Zimbone S Infantino IR. VDAC1-targeted NHK1 peptide recovers mitochondrial dysfunction counteracting amyloid-β oligomers toxicity in Alzheimer's disease. Aging Cell. (2025) 24:e70069. doi: 10.1111/acel.70069, PMID: 40223243 PMC12266788

[45] Karikari TK Ashton NJ Brinkmalm G Brum WS Benedet AL Montoliu-Gaya L. Blood phospho-tau in Alzheimer disease: analysis, interpretation, and clinical utility. Nat Rev Neurol. (2022) 18:400–18. doi: 10.1038/s41582-022-00665-2, PMID: 35585226

[46] Pinto TCC Machado L Bulgacov TM Rodrigues-Júnior AL Costa MLG Ximenes RCC. Is the Montreal cognitive assessment (MoCA) screening superior to the Mini-mental state examination (MMSE) in the detection of mild cognitive impairment (MCI) and Alzheimer's disease (AD) in the elderly? Int Psychogeriatr. (2019) 31:491–504. doi: 10.1017/s1041610218001370, PMID: 30426911

[47] Tokumitsu K Yasui-Furukori N Takeuchi J Yachimori K Sugawara N Terayama Y. The combination of MMSE with VSRAD and eZIS has greater accuracy for discriminating mild cognitive impairment from early Alzheimer's disease than MMSE alone. PLoS One. (2021) 16:e0247427. doi: 10.1371/journal.pone.0247427, PMID: 33617587 PMC7899318

[48] Ruan Z Pathak D Venkatesan Kalavai S Yoshii-Kitahara A Muraoka S Bhatt N. Alzheimer's disease brain-derived extracellular vesicles spread tau pathology in interneurons. Brain. (2021) 144:288–309. doi: 10.1093/brain/awaa376, PMID: 33246331 PMC7880668

[49] Fonte C Smania N Pedrinolla A Munari D Gandolfi M Picelli A. Comparison between physical and cognitive treatment in patients with MCI and Alzheimer's disease. Aging (Albany NY). (2019) 11:3138–55. doi: 10.18632/aging.101970, PMID: 31127076 PMC6555450

[50] Chou YH Sundman M Ton That V Green J Trapani C. Cortical excitability and plasticity in Alzheimer's disease and mild cognitive impairment: a systematic review and meta-analysis of transcranial magnetic stimulation studies. Ageing Res Rev. (2022) 79:101660. doi: 10.1016/j.arr.2022.101660, PMID: 35680080 PMC9707650

[51] Chou YH Ton That V Sundman M. A systematic review and meta-analysis of rTMS effects on cognitive enhancement in mild cognitive impairment and Alzheimer's disease. Neurobiol Aging. (2020) 86:1–10. doi: 10.1016/j.neurobiolaging.2019.08.020, PMID: 31783330 PMC6995441

[52] Sørensen L Igel C Liv Hansen N Osler M Lauritzen M Rostrup E. Early detection of Alzheimer's disease using MRI hippocampal texture. Hum Brain Mapp. (2016) 37:1148–61. doi: 10.1002/hbm.23091, PMID: 26686837 PMC6867374

[53] Feng Q Ding Z. MRI radiomics classification and prediction in Alzheimer's disease and mild cognitive impairment: a review. Curr Alzheimer Res. (2020) 17:297–309. doi: 10.2174/1567205017666200303105016, PMID: 32124697

[54] You SH Kim B Kim I Yang KS Kim KM Kim BK. Integrative MR imaging interpretation in cognitive impairment with Alzheimer's disease, Small vessel disease, and Glymphatic function-related MR parameters. Acad Radiol. (2025) 32:932–50. doi: 10.1016/j.acra.2024.08.034, PMID: 39294052

[55] Sørensen L Igel C Pai A Balas I Anker C Lillholm M. Differential diagnosis of mild cognitive impairment and Alzheimer's disease using structural MRI cortical thickness, hippocampal shape, hippocampal texture, and volumetry. Neuroimage Clin. (2016) 13:470–82. doi: 10.1016/j.nicl.2016.11.025, PMID: 28119818 PMC5237821

[56] Fekete M Varga P Ungvari Z Fekete JT Buda A Szappanos Á. The role of the Mediterranean diet in reducing the risk of cognitive impairement, dementia, and Alzheimer's disease: a meta-analysis. Geroscience. (2025) 47:3111–30. doi: 10.1007/s11357-024-01488-3, PMID: 39797935 PMC12181514

[57] Feng W Halm-Lutterodt NV Tang H Mecum A Mesregah MK Ma Y. Automated MRI-based deep learning model for detection of Alzheimer's disease process. Int J Neural Syst. (2020) 30:2050032. doi: 10.1142/S012906572050032, PMID: 32498641

[58] Ding Y Zhao K Che T Du K Sun H Liu S. Quantitative radiomic features as new biomarkers for Alzheimer's disease: an amyloid PET study. Cereb Cortex. (2021) 31:3950–61. doi: 10.1093/cercor/bhab061, PMID: 33884402

[59] Clark CM Pontecorvo MJ Beach TG Bedell BJ Coleman RE Doraiswamy PM. Cerebral PET with florbetapir compared with neuropathology at autopsy for detection of neuritic amyloid-β plaques: a prospective cohort study. Lancet Neurol. (2012) 11:669–78. doi: 10.1016/s1474-4422(12)70142-4, PMID: 22749065

[60] Perani D Iaccarino L Lammertsma AA Windhorst AD Edison P Boellaard R. A new perspective for advanced positron emission tomography-based molecular imaging in neurodegenerative proteinopathies. Alzheimers Dement. (2019) 15:1081–103. doi: 10.1016/j.jalz.2019.02.004, PMID: 31230910

[61] Thijssen EH La Joie R Strom A. Plasma phosphorylated tau 217 and phosphorylated tau 181 as biomarkers in Alzheimer's disease and frontotemporal lobar degeneration: a retrospective diagnostic performance study. Lancet Neurol. (2021) 20:739–52. doi: 10.1016/s1474-4422(21)00214-3, PMID: 34418401 PMC8711249

[62] Donaghy PC Firbank MJ Thomas AJ Lloyd J Petrides G Barnett N. Clinical and imaging correlates of amyloid deposition in dementia with Lewy bodies. Mov Disord. (2018) 33:1130–8. doi: 10.1002/mds.27403, PMID: 29672930 PMC6175485

[63] Walker L Simpson H Thomas AJ Attems J. Prevalence, distribution, and severity of cerebral amyloid angiopathy differ between Lewy body diseases and Alzheimer's disease. Acta Neuropathol Commun. (2024) 12:28. doi: 10.1186/s40478-023-01714-7, PMID: 38360761 PMC10870546

[64] Boccalini C Ribaldi F Hristovska I Arnone A Peretti DE Mu L. The impact of tau deposition and hypometabolism on cognitive impairment and longitudinal cognitive decline. Alzheimers Dement. (2024) 20:221–33. doi: 10.1002/alz.13355, PMID: 37555516 PMC10916991

[65] Gérard T Colmant L Malotaux V Salman Y Huyghe L Quenon L. Tau PET imaging with [18F]MK-6240: limited affinity for primary tauopathies and high specificity for Alzheimer's disease. Eur J Neurol. (2025) 32:e70068. doi: 10.1111/ene.70068, PMID: 39957301 PMC11831001

[66] Zhong X Wang Q Yang M Lin G Yao K Wu Z. Plasma p-tau217 and p-tau217/Aβ1-42 are effective biomarkers for identifying CSF- and PET imaging-diagnosed Alzheimer's disease: insights for research and clinical practice. Alzheimers Dement. (2025) 21:e14536. doi: 10.1002/alz.14536, PMID: 39887504 PMC11848202

[67] Chong JR Ashton NJ Karikari TK Tanaka T Schöll M Zetterberg H. Blood-based high sensitivity measurements of beta-amyloid and phosphorylated tau as biomarkers of Alzheimer's disease: a focused review on recent advances. J Neurol Neurosurg Psychiatry. (2021) 92:1231–41. doi: 10.1136/jnnp-2021-327370, PMID: 34510001

[68] Kaeser SA Häsler LM Lambert M Bergmann C Bottelbergs A Theunis C. CSF p-tau increase in response to aβ-type and Danish-type cerebral amyloidosis and in the absence of neurofibrillary tangles. Acta Neuropathol. (2022) 143:287–90. doi: 10.1007/s00401-021-02400-5, PMID: 34961894 PMC8742811

[69] Largent EA Wexler A Karlawish J. The future is P-tau-anticipating direct-to-consumer Alzheimer disease blood tests. JAMA Neurol. (2021) 78:379–80. doi: 10.1001/jamaneurol.2020.4835, PMID: 33393982 PMC8035155

[70] Palmqvist S Janelidze S Quiroz YT Zetterberg H Lopera F Stomrud E. Discriminative accuracy of plasma Phospho-tau217 for Alzheimer disease vs other neurodegenerative disorders. JAMA. (2020) 324:772–81. doi: 10.1001/jama.2020.12134, PMID: 32722745 PMC7388060

[71] Jack CR Jr Bennett DA Blennow K Carrillo MC Dunn B Haeberlein SB. NIA-AA research framework: toward a biological definition of Alzheimer's disease. Alzheimers Dement. (2018) 14:535–62. doi: 10.1016/j.jalz.2018.02.018, PMID: 29653606 PMC5958625

[72] Ayton S Janelidze S Roberts B Palmqvist S Kalinowski P Diouf I. Acute phase markers in CSF reveal inflammatory changes in Alzheimer's disease that intersect with pathology, APOE ε4, sex and age. Prog Neurobiol. (2021) 198:101904. doi: 10.1016/j.pneurobio.2020.101904 32882319

[73] Hampel H Cummings J Blennow K Gao P Jack CR Jr Vergallo A. Developing the ATX(N) classification for use across the Alzheimer disease continuum. Nat Rev Neurol. (2021) 17:580–9. doi: 10.1038/s41582-021-00520-w, PMID: 34239130

[74] de Wolf F Ghanbari M Licher S McRae-McKee K Gras L Weverling GJ. Plasma tau, neurofilament light chain and amyloid-β levels and risk of dementia; a population-based cohort study. Brain. (2020) 143:1220–32. doi: 10.1093/brain/awaa054, PMID: 32206776 PMC7174054

[75] Palmqvist S Insel PS Stomrud E Janelidze S Zetterberg H Brix B. Cerebrospinal fluid and plasma biomarker trajectories with increasing amyloid deposition in Alzheimer's disease. EMBO Mol Med. (2019) 11:e11170. doi: 10.15252/emmm.201911170, PMID: 31709776 PMC6895602

[76] Inyushin M Zayas-Santiago A Rojas L Kucheryavykh L. On the role of platelet-generated amyloid Beta peptides in certain amyloidosis health complications. Front Immunol. (2020) 11:571083. doi: 10.3389/fimmu.2020.571083, PMID: 33123145 PMC7567018

[77] Schindler SE Bollinger JG Ovod V Mawuenyega KG Li Y Gordon BA. High-precision plasma β-amyloid 42/40 predicts current and future brain amyloidosis. Neurology. (2019) 93:e1647–59. doi: 10.1212/wnl.0000000000008081, PMID: 31371569 PMC6946467

[78] Nakamura A Kaneko N Villemagne VL. High performance plasma amyloid-β biomarkers for Alzheimer's disease. Nature. (2018) 554:249–54. doi: 10.1038/nature25456 29420472

[79] Willumsen N Poole T Nicholas JM Fox NC Ryan NS Lashley T. Variability in the type and layer distribution of cortical aβ pathology in familial Alzheimer's disease. Brain Pathol. (2022) 32:e13009. doi: 10.1111/bpa.13009, PMID: 34319632 PMC9048809

[80] Delvaux E Bentley K Stubbs V Sabbagh M Coleman PD. Differential processing of amyloid precursor protein in brain and in peripheral blood leukocytes. Neurobiol Aging. (2013) 34:1680–6. doi: 10.1016/j.neurobiolaging.2012.12.004, PMID: 23298733 PMC3598628

[81] Choi SI Lee B Woo JH Jeong JB Jun I Kim EK. APP processing and metabolism in corneal fibroblasts and epithelium as a potential biomarker for Alzheimer's disease. Exp Eye Res. (2019) 182:167–74. doi: 10.1016/j.exer.2019.03.012, PMID: 30930125

[82] Ercan-Herbst E Ehrig J Schöndorf DC Behrendt A Klaus B Gomez Ramos B. A post-translational modification signature defines changes in soluble tau correlating with oligomerization in early stage Alzheimer's disease brain. Acta Neuropathol Commun. (2019) 7:192. doi: 10.1186/s40478-019-0823-2, PMID: 31796124 PMC6892178

[83] Barthélemy NR Horie K Sato C Bateman RJ. Blood plasma phosphorylated-tau isoforms track CNS change in Alzheimer's disease. J Exp Med. (2020) 217:e20200861. doi: 10.1084/jem.20200861 32725127 PMC7596823

[84] Leuzy A Janelidze S Mattsson-Carlgren N Palmqvist S Jacobs D Cicognola C. Comparing the clinical utility and diagnostic performance of CSF P-Tau181, P-Tau217, and P-Tau231 assays. Neurology. (2021) 97:e1681–94. doi: 10.1212/wnl.0000000000012727, PMID: 34493616 PMC8605616

[85] Mielke MM Frank RD Dage JL Jeromin A Ashton NJ Blennow K. Comparison of plasma phosphorylated tau species with amyloid and tau positron emission tomography, neurodegeneration, vascular pathology, and cognitive outcomes. JAMA Neurol. (2021) 78:1108–17. doi: 10.1001/jamaneurol.2021.2293, PMID: 34309632 PMC8314178

[86] Gu J Xu W Jin N Li L Zhou Y Chu D. Truncation of tau selectively facilitates its pathological activities. J Biol Chem. (2020) 295:13812–28. doi: 10.1074/jbc.RA120.012587, PMID: 32737201 PMC7535906

[87] Blennow K Chen C Cicognola C Wildsmith KR Manser PT Bohorquez SMS. Cerebrospinal fluid tau fragment correlates with tau PET: a candidate biomarker for tangle pathology. Brain. (2020) 143:650–60. doi: 10.1093/brain/awz346, PMID: 31834365 PMC7009597

[88] LoBue C Stopschinski BE Saez Calveras N Douglas PM Huebinger R Cullum CM. Blood markers in relation to a history of traumatic brain injury across stages of cognitive impairment in a diverse cohort. J Alzheimer's Dis. (2024) 97:345–58. doi: 10.3233/jad-231027, PMID: 38143366 PMC10947497

[89] Antonell A Tort-Merino A Ríos J Balasa M Borrego-Écija S Auge JM. Synaptic, axonal damage and inflammatory cerebrospinal fluid biomarkers in neurodegenerative dementias. Alzheimers Dement. (2020) 16:262–72. doi: 10.1016/j.jalz.2019.09.001, PMID: 31668967

[90] Zetterberg H. Review: tau in biofluids - relation to pathology, imaging and clinical features. Neuropathol Appl Neurobiol. (2017) 43:194–9. doi: 10.1111/nan.12378, PMID: 28054371

[91] Oh HS Urey DY Karlsson L Zhu Z Shen Y Farinas A. A cerebrospinal fluid synaptic protein biomarker for prediction of cognitive resilience versus decline in Alzheimer's disease. Nat Med. (2025) 31:1592–603. doi: 10.1038/s41591-025-03565-2, PMID: 40164724 PMC12092275

[92] Öhrfelt A Dumurgier J Zetterberg H Vrillon A Ashton NJ Kvartsberg H. Full-length and C-terminal neurogranin in Alzheimer's disease cerebrospinal fluid analyzed by novel ultrasensitive immunoassays. Alzheimer's Res Ther. (2020) 12:168. doi: 10.1186/s13195-020-00748-6, PMID: 33353563 PMC7756958

[93] Mavroudis IA Petridis F Chatzikonstantinou S Kazis D. A meta-analysis on CSF neurogranin levels for the diagnosis of Alzheimer's disease and mild cognitive impairment. Aging Clin Exp Res. (2020) 32:1639–46. doi: 10.1007/s40520-019-01326-z, PMID: 31463927

[94] Milà-Alomà M Brinkmalm A Ashton NJ Kvartsberg H Shekari M Operto G. CSF synaptic biomarkers in the preclinical stage of Alzheimer disease and their association with MRI and PET: a cross-sectional study. Neurology. (2021) 97:e2065–78. doi: 10.1212/wnl.0000000000012853, PMID: 34556565 PMC8610620

[95] Tarawneh R D'Angelo G Crimmins D. Diagnostic and prognostic utility of the synaptic marker Neurogranin in Alzheimer disease. JAMA Neurol. (2016) 73:561–71. doi: 10.1001/jamaneurol.2016.0086 27018940 PMC4861689

[96] De Vos A Jacobs D Struyfs H. C-terminal neurogranin is increased in cerebrospinal fluid but unchanged in plasma in Alzheimer's disease. Alzheimers Dement. (2015) 11:1461–9. doi: 10.1016/j.jalz.2015.05.012, PMID: 26092348

[97] He M Sun L Cao W Yin C Sun W Liu P. Association between plasma exosome neurogranin and brain structure in patients with Alzheimer's disease: a protocol study. BMJ Open. (2020) 10:e036990. doi: 10.1136/bmjopen-2020-036990, PMID: 32801201 PMC7430441

[98] Schindler SE Karikari TK Ashton NJ Henson RL Yarasheski KE West T. Effect of race on prediction of brain amyloidosis by plasma Aβ42/Aβ40, phosphorylated tau, and neurofilament light. Neurology. (2022) 99:e245–57. doi: 10.1212/WNL.0000000000200358, PMID: 35450967 PMC9302933

[99] Lim JK Li QX He Z Vingrys AJ Wong VH Currier N. The eye as a biomarker for Alzheimer's disease. Front Neurosci. (2016) 10:536. doi: 10.3389/fnins.2016.00536, PMID: 27909396 PMC5112261

[100] Patton N Aslam T Macgillivray T Pattie A Deary IJ Dhillon B. Retinal vascular image analysis as a potential screening tool for cerebrovascular disease: a rationale based on homology between cerebral and retinal microvasculatures. J Anat. (2005) 206:319–48. doi: 10.1111/j.1469-7580.2005.00395.x, PMID: 15817102 PMC1571489

[101] Gaire BP Koronyo Y Fuchs DT Shi H Rentsendorj A Danziger R. Alzheimer's disease pathophysiology in the retina. Prog Retin Eye Res. (2024) 101:101273. doi: 10.1016/j.preteyeres.2024.101273, PMID: 38759947 PMC11285518

[102] Yücel YH Zhang Q Weinreb RN Kaufman PL Gupta N. Effects of retinal ganglion cell loss on magno-, parvo-, koniocellular pathways in the lateral geniculate nucleus and visual cortex in glaucoma. Prog Retin Eye Res. (2003) 22:465–81. doi: 10.1016/s1350-9462(03)00026-0, PMID: 12742392

[103] Gupta VB Chitranshi N den Haan J Mirzaei M You Y Lim JK. Retinal changes in Alzheimer's disease- integrated prospects of imaging, functional and molecular advances. Prog Retin Eye Res. (2021) 82:100899. doi: 10.1016/j.preteyeres.2020.100899, PMID: 32890742

[104] Marsden IT Minamide LS Bamburg JR. Amyloid-β-induced amyloid-β secretion: a possible feed-forward mechanism in Alzheimer's disease. J Alzheimer's Dis. (2011) 24:681–91. doi: 10.3233/jad-2011-101899, PMID: 21297255 PMC4447202

[105] Koronyo Y Salumbides BC Black KL Koronyo-Hamaoui M. Alzheimer's disease in the retina: imaging retinal aβ plaques for early diagnosis and therapy assessment. Neurodegener Dis. (2012) 10:285–93. doi: 10.1159/000335154, PMID: 22343730

[106] Brettschneider J Del Tredici K Lee VM Trojanowski JQ. Spreading of pathology in neurodegenerative diseases: a focus on human studies. Nat Rev Neurosci. (2015) 16:109–20. doi: 10.1038/nrn3887, PMID: 25588378 PMC4312418

[107] Gibbons GS Lee VMY Trojanowski JQ. Mechanisms of cell-to-cell transmission of pathological tau: a review. JAMA Neurol. (2019) 76:101–8. doi: 10.1001/jamaneurol.2018.2505, PMID: 30193298 PMC6382549

[108] Hanger DP Byers HL Wray S Leung KY Saxton MJ Seereeram A. Novel phosphorylation sites in tau from Alzheimer brain support a role for casein kinase 1 in disease pathogenesis. J Biol Chem. (2007) 282:23645–54. doi: 10.1074/jbc.M703269200, PMID: 17562708

[109] Chitranshi N Dheer Y Mirzaei M Wu Y Salekdeh GH Abbasi M. Loss of Shp2 rescues BDNF/TrkB signaling and contributes to improved retinal ganglion cell neuroprotection. Mol Ther. (2019) 27:424–41. doi: 10.1016/j.ymthe.2018.09.019, PMID: 30341011 PMC6369445

[110] Chiasseu M Alarcon-Martinez L Belforte N Quintero H Dotigny F Destroismaisons L. Tau accumulation in the retina promotes early neuronal dysfunction and precedes brain pathology in a mouse model of Alzheimer's disease. Mol Neurodegener. (2017) 12:58. doi: 10.1186/s13024-017-0199-3, PMID: 28774322 PMC5543446

[111] Gupta VK Chitranshi N Gupta VB Golzan M Dheer Y Wall RV. Amyloid β accumulation and inner retinal degenerative changes in Alzheimer's disease transgenic mouse. Neurosci Lett. (2016) 623:52–6. doi: 10.1016/j.neulet.2016.04.059, PMID: 27133194

[112] Carazo-Barrios L Cabrera-Maestre A Alba-Linero C Gutiérrez-Bedmar M Garzón-Maldonado FJ Serrano V. Retinal neurodegeneration measured with optical coherence tomography and neuroimaging in Alzheimer disease: a systematic review. J Neuroophthalmol. (2023) 43:116–25. doi: 10.1097/wno.0000000000001673, PMID: 36255105

[113] López-de-Eguileta A Lage C López-García S Pozueta A García-Martínez M Kazimierczak M. Ganglion cell layer thinning in prodromal Alzheimer's disease defined by amyloid PET. Alzheimers Dement. (2019) 5:570–8. doi: 10.1016/j.trci.2019.08.008, PMID: 31650013 PMC6804512

[114] Ko F Muthy ZA Gallacher J Sudlow C Rees G Yang Q. Association of Retinal Nerve Fiber Layer Thinning with Current and Future Cognitive Decline: a study using optical coherence tomography. JAMA Neurol. (2018) 75:1198–205. doi: 10.1001/jamaneurol.2018.1578, PMID: 29946685 PMC6233846

[115] Cox KH Pipingas A Scholey AB. Investigation of the effects of solid lipid curcumin on cognition and mood in a healthy older population. J Psychopharmacol. (2015) 29:642–51. doi: 10.1177/0269881114552744 25277322

[116] Bader I Groot C Tan HS Milongo J-MA Haan J den Verberk IMW. Rationale and design of the BeyeOMARKER study: prospective evaluation of blood- and eye-based biomarkers for early detection of Alzheimer's disease pathology in the eye clinic. Alzheimer's Res Ther. (2024) 16:190. doi: 10.1186/s13195-024-01545-1, PMID: 39169442 PMC11340081

[117] Koronyo-Hamaoui M Koronyo Y Ljubimov AV Miller CA Ko MHK Black KL. Identification of amyloid plaques in retinas from Alzheimer's patients and noninvasive in vivo optical imaging of retinal plaques in a mouse model. NeuroImage. (2011) 54:S204–17. doi: 10.1016/j.neuroimage.2010.06.020, PMID: 20550967 PMC2991559

[118] Kwak DE Ko T Koh HS Ji YW Shin J Kim K. Alterations of aqueous humor aβ levels in aβ-infused and transgenic mouse models of Alzheimer disease. PLoS One. (2020) 15:e0227618. doi: 10.1371/journal.pone.0227618, PMID: 31923257 PMC6953883

[119] Bai J Wan Z Wang M Wu X Wang T Zhang Y. Association of cognitive function with Neurofilament light chain in the aqueous humor of human eye. Front Aging Neurosci. (2022) 14:1027705. doi: 10.3389/fnagi.2022.1027705, PMID: 36408096 PMC9671656

[120] Vig V Garg I Tuz-Zahra F Xu J Tripodis Y Nicks R. Vitreous humor biomarkers reflect pathological changes in the brain for Alzheimer's disease and chronic traumatic encephalopathy. J Alzheimer's Dis. (2023) 93:1181–93. doi: 10.3233/jad-230167, PMID: 37182888 PMC10258881

[121] Wright LM Stein TD Jun G Chung J McConnell K Fiorello M. Association of Cognitive Function with amyloid-β and tau proteins in the vitreous humor. J Alzheimer's Dis. (2019) 68:1429–38. doi: 10.3233/jad-181104, PMID: 30856114 PMC6850770

[122] Sampani K Ness S Tuz-Zahra F Aytan N Spurlock EE Alluri S. Neurodegenerative biomarkers in different chambers of the eye relative to plasma: an agreement validation study. Alzheimer's Res Ther. (2024) 16:192. doi: 10.1186/s13195-024-01556-y, PMID: 39187891 PMC11346268

[123] Madeira MH Boia R Santos PF Ambrósio AF Santiago AR. Contribution of microglia-mediated neuroinflammation to retinal degenerative diseases. Mediat Inflamm. (2015) 2015:673090. doi: 10.1155/2015/673090, PMID: 25873768 PMC4385698

[124] García-Bermúdez MY Vohra R Freude K. Potential retinal biomarkers in Alzheimer's disease. Int J Mol Sci. (2023) 24. doi: 10.3390/ijms242115834 PMC10649108 37958816

[125] Lee S Kim E Moon CE Park C Lim JW Baek M. Amplified fluorogenic immunoassay for early diagnosis and monitoring of Alzheimer's disease from tear fluid. Nat Commun. (2023) 14:8153. doi: 10.1038/s41467-023-43995-5, PMID: 38071202 PMC10710446

[126] Yin Q Ji X Lv R Pei JJ du Y Shen C. Targetting exosomes as a new biomarker and therapeutic approach for Alzheimer's disease. Clin Interv Aging. (2020) 15:195–205. doi: 10.2147/cia.S240400, PMID: 32103922 PMC7025655

[127] Poudineh M Maikawa CL Ma EY Pan J Mamerow D Hang Y. A fluorescence sandwich immunoassay for the real-time continuous detection of glucose and insulin in live animals. Nat Biomed Eng. (2021) 5:53–63. doi: 10.1038/s41551-020-00661-1, PMID: 33349659 PMC7856282

[128] Shin MK Ji YW Moon CE Lee H Kang B Jinn WS. Matrix metalloproteinase 9-activatable peptide-conjugated hydrogel-based fluorogenic intraocular-lens sensor. Biosens Bioelectron. (2020) 162:112254. doi: 10.1016/j.bios.2020.112254, PMID: 32392157

[129] Kim S Kim G Ji YW Moon CE Jung Y Lee HK. Real-time and label-free biosensing using moiré pattern generated by bioresponsive hydrogel. Bioact Mater. (2023) 23:383–93. doi: 10.1016/j.bioactmat.2022.11.010, PMID: 36474658 PMC9712824

[130] Cummings J Lee G Zhong K Fonseca J Taghva K. Alzheimer's disease drug development pipeline: 2021. Alzheimers Dement. (2021) 7:e12179. doi: 10.1002/trc2.12179, PMID: 34095440 PMC8145448

[131] Xu MR Dai RF Wei QQ Wang J Feng YY Hu Y. Urinary AD7c-NTP evaluates cognition impairment and differentially diagnoses AD and MCI. Am J Alzheimers Dis Other Dement. (2022) 37:15333175221115247. doi: 10.1177/15333175221115247, PMID: 35833655 PMC10581138

[132] de la Monte SM Wands JR. The AD7C-NTP neuronal thread protein biomarker for detecting Alzheimer's disease. Front Biosci. (2002) 7:d989–96. doi: 10.2741/monte, PMID: 11897561

[133] Ghanbari H Ghanbari K Beheshti I Munzar M Vasauskas A Averback P. Biochemical assay for AD7C-NTP in urine as an Alzheimer's disease marker. J Clin Lab Anal. (1998) 12:285–8. doi: 10.1002/(sici)1098-2825(1998)12:5<285::aid-jcla6>3.0.co;2-5, PMID: 9773959 PMC6808140

[134] Chen Y Shi S Zhang J. Diagnostic value of AD7C-NTP for patients with mild cognitive impairment due to Alzheimer's disease. Zhonghua Yi Xue Za Zhi. (2014) 94:1613–7. 25152281

[135] Jiao B Zhang S Bei Y Bu G Yuan L Zhu Y. A detection model for cognitive dysfunction based on volatile organic compounds from a large Chinese community cohort. Alzheimers Dement. (2023) 19:4852–62. doi: 10.1002/alz.13053, PMID: 37032600

[136] Bach JP Gold M Mengel D Hattesohl A Lubbe D Schmid S. Measuring compounds in exhaled air to detect Alzheimer's disease and Parkinson's disease. PLoS One. (2015) 10:e0132227. doi: 10.1371/journal.pone.0132227, PMID: 26168044 PMC4500505

[137] Jia L Yang J Zhu M Pang Y Wang Q Wei Q. A metabolite panel that differentiates Alzheimer's disease from other dementia types. Alzheimers Dement. (2022) 18:1345–56. doi: 10.1002/alz.12484, PMID: 34786838 PMC9545206

[138] Dong X Tang J Ren Y Chen X. Development of a HPLC-FL method to determine benzaldehyde after derivatization with N-acetylhydrazine acridone and its application for determination of semicarbazide-sensitive amine oxidase activity in human serum. RSC Adv. (2019) 9:6717–23. doi: 10.1039/c8ra10004g, PMID: 35518507 PMC9061080

[139] Tsou HH Hsu WC Fuh JL Chen SP Liu TY Wang HT. Alterations in Acrolein metabolism contribute to Alzheimer's disease. J Alzheimer's Dis. (2018) 61:571–80. doi: 10.3233/jad-170736 29226874

[140] Chirila FV Xu G Fontaine D Kern G Khan TK Brandt J. Morphometric imaging biomarker identifies Alzheimer's disease even among mixed dementia patients. Sci Rep. (2022) 12:17675. doi: 10.1038/s41598-022-21796-y, PMID: 36319674 PMC9626495

[141] Chirila FV Khan TK Alkon DL. Fibroblast aggregation rate converges with validated peripheral biomarkers for Alzheimer's disease. J Alzheimer's Dis. (2014) 42:1279–94. doi: 10.3233/jad-140672, PMID: 25024330

[142] Khan TK Alkon DL. Early diagnostic accuracy and pathophysiologic relevance of an autopsy-confirmed Alzheimer's disease peripheral biomarker. Neurobiol Aging. (2010) 31:889–900. doi: 10.1016/j.neurobiolaging.2008.07.010, PMID: 18760507

[143] Ferrer I Blanco R Carmona M Ribera R Goutan E Puig B. Phosphorylated map kinase (ERK1, ERK2) expression is associated with early tau deposition in neurones and glial cells, but not with increased nuclear DNA vulnerability and cell death, in Alzheimer disease, pick's disease, progressive supranuclear palsy and corticobasal degeneration. Brain Pathol. (2001) 11:144–58. doi: 10.1111/j.1750-3639.2001.tb00387.x, PMID: 11303790 PMC8098611

[144] Khan TK Alkon DL. An internally controlled peripheral biomarker for Alzheimer's disease: Erk1 and Erk2 responses to the inflammatory signal bradykinin. Proc Natl Acad Sci USA. (2006) 103:13203–7. doi: 10.1073/pnas.0605411103, PMID: 16920798 PMC1559777

[145] Indrigo M Morella I Orellana D d'Isa R Papale A Parra R. Nuclear ERK1/2 signaling potentiation enhances neuroprotection and cognition via Importinα1/KPNA2. EMBO Mol Med. (2023) 15:e15984. doi: 10.15252/emmm.202215984, PMID: 37792911 PMC10630888

[146] Giacomucci G Mazzeo S Bagnoli S Ingannato A Leccese D Berti V. Plasma neurofilament light chain as a biomarker of Alzheimer's disease in subjective cognitive decline and mild cognitive impairment. J Neurol. (2022) 269:4270–80. doi: 10.1007/s00415-022-11055-5, PMID: 35288777 PMC9293849

[147] Palmqvist S Tideman P Mattsson-Carlgren N Schindler SE Smith R Ossenkoppele R. Blood biomarkers to detect Alzheimer disease in primary care and secondary care. JAMA. (2024) 332:1245–57. doi: 10.1001/jama.2024.13855, PMID: 39068545 PMC11284636

[148] Bai B Vanderwall D Li Y Wang X Poudel S Wang H. Proteomic landscape of Alzheimer's disease: novel insights into pathogenesis and biomarker discovery. Mol Neurodegener. (2021) 16:55. doi: 10.1186/s13024-021-00474-z, PMID: 34384464 PMC8359598

[149] Aerqin Q Wang ZT Wu KM He XY Dong Q Yu JT. Omics-based biomarkers discovery for Alzheimer's disease. Cell Mol Life Sci. (2022) 79:585. doi: 10.1007/s00018-022-04614-6, PMID: 36348101 PMC11803048

[150] Fattahi F Asadi MR Abed S Kouchakali G Kazemi M Mansoori Derakhshan S. Blood-based microRNAs as the potential biomarkers for Alzheimer's disease: evidence from a systematic review. Metab Brain Dis. (2024) 40:44. doi: 10.1007/s11011-024-01431-7, PMID: 39607566

[151] Horgusluoglu E Neff R Song WM Wang M Wang Q Arnold M. Integrative metabolomics-genomics approach reveals key metabolic pathways and regulators of Alzheimer's disease. Alzheimers Dement. (2022) 18:1260–78. doi: 10.1002/alz.12468, PMID: 34757660 PMC9085975

[152] Duan S Cai T Liu F Li Y Yuan H Yuan W. Automatic offline-capable smartphone paper-based microfluidic device for efficient biomarker detection of Alzheimer's disease. Anal Chim Acta. (2024) 1308:342575. doi: 10.1016/j.aca.2024.342575, PMID: 38740448

[153] Sequeira-Antunes B Ferreira HA. Urinary biomarkers and point-of-care urinalysis devices for early diagnosis and Management of Disease: a review. Biomedicine. (2023) 11. doi: 10.3390/biomedicines11041051 PMC10135468 37189669

[154] Talebi M Esmaeeli H Talebi M Farkhondeh T Samarghandian S. A concise overview of biosensing Technologies for the Detection of Alzheimer's disease biomarkers. Curr Pharm Biotechnol. (2022) 23:634–44. doi: 10.2174/2666796702666210709122407, PMID: 34250871

[155] Mahaman YAR Embaye KS Huang F Li L Zhu F Wang JZ. Biomarkers used in Alzheimer's disease diagnosis, treatment, and prevention. Ageing Res Rev. (2022) 74:101544. doi: 10.1016/j.arr.2021.101544, PMID: 34933129

[156] Liss JL Seleri Assunção S Cummings J Atri A Geldmacher DS Candela SF. Practical recommendations for timely, accurate diagnosis of symptomatic Alzheimer's disease (MCI and dementia) in primary care: a review and synthesis. J Intern Med. (2021) 290:310–34. doi: 10.1111/joim.13244, PMID: 33458891 PMC8359937

[157] De Marchi F Vignaroli F Mazzini L Comi C Tondo G. New insights into the relationship between nutrition and Neuroinflammation in Alzheimer's disease: preventive and therapeutic perspectives. CNS Neurol Disord Drug Targets. (2024) 23:614–27. doi: 10.2174/1871527322666230608110201, PMID: 37291780

[158] Babiloni C Blinowska K Bonanni L Cichocki A De Haan W Del Percio C. What electrophysiology tells us about Alzheimer's disease: a window into the synchronization and connectivity of brain neurons. Neurobiol Aging. (2020) 85:58–73. doi: 10.1016/j.neurobiolaging.2019.09.008, PMID: 31739167

[159] Marvi F Chen YH Sawan M. Alzheimer's disease diagnosis in the preclinical stage: Normal aging or dementia. IEEE Rev Biomed Eng. (2025) 18:74–92. doi: 10.1109/rbme.2024.3376835, PMID: 38478432

[160] Bondi MW Edmonds EC Salmon DP. Alzheimer's disease: past, present, and future. J Int Neuropsychol Soc. (2017) 23:818–31. doi: 10.1017/s135561771700100x, PMID: 29198280 PMC5830188

[161] Ma C Hong F Yang S. Amyloidosis in Alzheimer's disease: pathogeny, etiology, and related therapeutic directions. Molecules. (2022) 27. doi: 10.3390/molecules27041210 PMC8876037 35209007

[162] Ghasemi F Hormozi-Nezhad MR Mahmoudi M. Label-free detection of β-amyloid peptides (Aβ40 and Aβ42): a colorimetric sensor array for plasma monitoring of Alzheimer's disease. Nanoscale. (2018) 10:6361–8. doi: 10.1039/c8nr00195b, PMID: 29561053

[163] Altug H Oh SH Maier SA Homola J. Advances and applications of nanophotonic biosensors. Nat Nanotechnol. (2022) 17:5–16. doi: 10.1038/s41565-021-01045-5, PMID: 35046571

[164] Poudel P Park S Castellano G. Recent advances in the treatment of Alzheimer's disease using nanoparticle-based drug delivery systems. Pharmaceutics. (2022) 14. doi: 10.3390/pharmaceutics14040835 PMC9026997 35456671

[165] Sperling RA Jack CR Aisen PS. Testing the right target and right drug at the right stage. Sci Transl Med. (2011) 3:111cm33. doi: 10.1126/scitranslmed.3002609, PMID: 22133718 PMC3752906

[166] Wang J Shangguan P Chen X Zhong Y Lin M He M. A one-two punch targeting reactive oxygen species and fibril for rescuing Alzheimer's disease. Nat Commun. (2024) 15:705. doi: 10.1038/s41467-024-44737-x, PMID: 38267418 PMC10808243

[167] Li J Sun C Tao W Cao Z Qian H Yang X. Photoinduced PEG deshielding from ROS-sensitive linkage-bridged block copolymer-based nanocarriers for on-demand drug delivery. Biomaterials. (2018) 170:147–55. doi: 10.1016/j.biomaterials.2018.04.015, PMID: 29674231

[168] Fan K Xi J Fan L Wang P Zhu C Tang Y. In vivo guiding nitrogen-doped carbon nanozyme for tumor catalytic therapy. Nat Commun. (2018) 9:1440. doi: 10.1038/s41467-018-03903-8, PMID: 29650959 PMC5897348

[169] Marenich AV Cramer CJ Truhlar DG. Universal solvation model based on solute electron density and on a continuum model of the solvent defined by the bulk dielectric constant and atomic surface tensions. J Phys Chem B. (2009) 113:6378–96. doi: 10.1021/jp810292n, PMID: 19366259

[170] Heo CH Sarkar AR Baik SH Jung TS Kim JJ Kang H. A quadrupolar two-photon fluorescent probe for in vivo imaging of amyloid-β plaques. Chem Sci. (2016) 7:4600–6. doi: 10.1039/c6sc00355a, PMID: 30155107 PMC6016450

[171] Fu H Cui M Zhao L Tu P Zhou K Dai J. Highly sensitive near-infrared fluorophores for in vivo detection of amyloid-β plaques in Alzheimer's disease. J Med Chem. (2015) 58:6972–83. doi: 10.1021/acs.jmedchem.5b00861, PMID: 26262759

[172] de Oliveira MS Balthazar ML D'Abreu A. MR imaging texture analysis of the corpus callosum and thalamus in amnestic mild cognitive impairment and mild Alzheimer disease. AJNR Am J Neuroradiol. (2011) 32:60–6. doi: 10.3174/ajnr.A2232 20966061 PMC7964940

[173] Chang CH Lin CH Lane HY. Machine learning and novel biomarkers for the diagnosis of Alzheimer's disease. Int J Mol Sci. (2021) 22. doi: 10.3390/ijms22052761 PMC7963160 33803217

[174] Wiest R Burren Y Hauf M Schroth G Pruessner J Zbinden M. Classification of mild cognitive impairment and Alzheimer disease using model-based MR and magnetization transfer imaging. AJNR Am J Neuroradiol. (2013) 34:740–6. doi: 10.3174/ajnr.A3307, PMID: 23064592 PMC7964474

[175] Chouhan N Khan A Shah JZ Hussnain M Khan MW. Deep convolutional neural network and emotional learning based breast cancer detection using digital mammography. Comput Biol Med. (2021) 132:104318. doi: 10.1016/j.compbiomed.2021.104318, PMID: 33744608

[176] Jin D Zhou B Han Y Ren J Han T Liu B. Generalizable, reproducible, and Neuroscientifically interpretable imaging biomarkers for Alzheimer's disease. Adv Sci. (2020) 7:2000675. doi: 10.1002/advs.202000675, PMID: 32714766 PMC7375255

[177] Grueso S Viejo-Sobera R. Machine learning methods for predicting progression from mild cognitive impairment to Alzheimer's disease dementia: a systematic review. Alzheimer's Res Ther. (2021) 13:162. doi: 10.1186/s13195-021-00900-w, PMID: 34583745 PMC8480074

[178] Mayo CD Garcia-Barrera MA Mazerolle EL Ritchie LJ Fisk JD Gawryluk JR. Relationship between DTI metrics and cognitive function in Alzheimer's disease. Front Aging Neurosci. (2018) 10:436. doi: 10.3389/fnagi.2018.00436, PMID: 30687081 PMC6333848

[179] Zhou X Chen Y Ip FCF Jiang Y Cao H Lv G. Deep learning-based polygenic risk analysis for Alzheimer's disease prediction. Commun Med. (2023) 3:49. doi: 10.1038/s43856-023-00269-x, PMID: 37024668 PMC10079691

[180] Lin RH Wang CC Tung CW. A machine learning classifier for predicting stable MCI patients using gene biomarkers. Int J Environ Res Public Health. (2022) 19. doi: 10.3390/ijerph19084839 PMC9025386 35457705

[181] Liu X Chen K Wu T Weidman D Lure F Li J. Use of multimodality imaging and artificial intelligence for diagnosis and prognosis of early stages of Alzheimer's disease. Transl Res. (2018) 194:56–67. doi: 10.1016/j.trsl.2018.01.001, PMID: 29352978 PMC5875456

[182] Kale M Wankhede N Pawar R Ballal S Kumawat R Goswami M. AI-driven innovations in Alzheimer's disease: integrating early diagnosis, personalized treatment, and prognostic modelling. Ageing Res Rev. (2024) 101:102497. doi: 10.1016/j.arr.2024.102497, PMID: 39293530

[183] Therriault J Schindler SE Salvadó G Pascoal TA Benedet AL Ashton NJ. Biomarker-based staging of Alzheimer disease: rationale and clinical applications. Nat Rev Neurol. (2024) 20:232–44. doi: 10.1038/s41582-024-00942-2, PMID: 38429551

[184] Therriault J Janelidze S Benedet AL Ashton NJ Arranz Martínez J Gonzalez-Escalante A. Diagnosis of Alzheimer's disease using plasma biomarkers adjusted to clinical probability. Nat Aging. (2024) 4:1529–37. doi: 10.1038/s43587-024-00731-y, PMID: 39533113 PMC11564087

[185] Hatcher H Stankeviciute S Learn C Qu AX. Regulatory, translational, and operational considerations for the incorporation of biomarkers in drug development. Ther Innov Regul Sci. (2025) 59:519–26. doi: 10.1007/s43441-025-00763-5 40057669

[186] Sharma R Gulati A Chopra K. Era of surrogate endpoints and accelerated approvals: a comprehensive review on applicability, uncertainties, and challenges from regulatory, payer, and patient perspectives. Eur J Clin Pharmacol. (2025) 81:605–23. doi: 10.1007/s00228-025-03822-w, PMID: 40080138

[187] Veziroglu EM Farhadi F Hasani N Nikpanah M Roschewski M Summers RM. Role of artificial intelligence in PET/CT imaging for Management of Lymphoma. Semin Nucl Med. (2023) 53:426–48. doi: 10.1053/j.semnuclmed.2022.11.003, PMID: 36870800

[188] Tae WS Ham BJ Pyun SB Kim BJ. Current clinical applications of structural MRI in neurological disorders. J Clin Neurol. (2025) 21:277–93. doi: 10.3988/jcn.2025.0185, PMID: 40635533 PMC12303675

[189] Wu J Shahid SS Lin Q Hone-Blanchet A Smith JL Risk BB. Multimodal magnetic resonance imaging reveals distinct sensitivity of hippocampal subfields in asymptomatic stage of Alzheimer's disease. Front Aging Neurosci. (2022) 14:901140. doi: 10.3389/fnagi.2022.901140, PMID: 36034141 PMC9413400

[190] Ruan D Sun L. Amyloid-β PET in Alzheimer's disease: a systematic review and Bayesian meta-analysis. Brain Behav. (2023) 13:e2850. doi: 10.1002/brb3.2850, PMID: 36573329 PMC9847612

[191] Ossenkoppele R Smith R Mattsson-Carlgren N Groot C Leuzy A Strandberg O. Accuracy of tau positron emission tomography as a prognostic marker in preclinical and prodromal Alzheimer disease: a head-to-head comparison against amyloid positron emission tomography and magnetic resonance imaging. JAMA Neurol. (2021) 78:961–71. doi: 10.1001/jamaneurol.2021.1858, PMID: 34180956 PMC8240013

